# Reaction of SARS-CoV-2 antibodies with other pathogens, vaccines, and food antigens

**DOI:** 10.3389/fimmu.2022.1003094

**Published:** 2022-09-23

**Authors:** Aristo Vojdani, Elroy Vojdani, Ashley L. Melgar, Joshua Redd

**Affiliations:** ^1^ Immunosciences Lab, Los Angeles, CA, United States; ^2^ Cyrex Laboratories, Limited Liability Company (LLC), Phoenix, AZ, United States; ^3^ Regenera Medical, Los Angeles, CA, United States; ^4^ Biological Sciences, UC Irvine, Irvine, CA, United States; ^5^ RedRiver Health and Wellness, South Jordan, UT, United States

**Keywords:** SARS-CoV-2, pathogens, vaccines, food antigens, molecular mimicry, antibodies

## Abstract

It has been shown that SARS-CoV-2 shares homology and cross-reacts with vaccines, other viruses, common bacteria and many human tissues. We were inspired by these findings, firstly, to investigate the reaction of SARS-CoV-2 monoclonal antibody with different pathogens and vaccines, particularly DTaP. Additionally, since our earlier studies have shown immune reactivity by antibodies made against pathogens and autoantigens towards different food antigens, we also studied cross-reaction between SARS-CoV-2 and common foods. For this, we reacted monoclonal and polyclonal antibodies against SARS-CoV-2 spike protein and nucleoprotein with 15 different bacterial and viral antigens and 2 different vaccines, BCG and DTaP, as well as with 180 different food peptides and proteins. The strongest reaction by SARS-CoV-2 antibodies were with DTaP vaccine antigen, *E. faecalis*, roasted almond, broccoli, soy, cashew, α+β casein and milk, pork, rice endochitinase, pineapple bromelain, and lentil lectin. Because the immune system tends to form immune responses towards the original version of an antigen that it has encountered, this cross-reactivity may have its advantages with regards to immunity against SARS-CoV-2, where the SARS-CoV-2 virus may elicit a “remembered” immune response because of its structural similarity to a pathogen or food antigen to which the immune system was previously exposed. Our findings indicate that cross-reactivity elicited by DTaP vaccines in combination with common herpesviruses, bacteria that are part of our normal flora such as *E. faecalis*, and foods that we consume on a daily basis should be investigated for possible cross-protection against COVID-19. Additional experiments would be needed to clarify whether or not this cross-protection is due to cross-reactive antibodies or long-term memory T and B cells in the blood.

## Introduction

The SARS-CoV-2 virus that is responsible for the COVID-19 pandemic is part of the family of coronaviruses that normally cause from mild to moderate upper-respiratory tract illness very similar to that of the common cold. However, the difference between SARS-CoV-2 and other viruses is its induction of serious illness with the involvement of multiple tissue abnormalities that may result in death (1).

When the body is exposed to different pathogens, it will launch an immune response, and afterwards the body will retain some disease-fighting cells called memory T and memory B cells. Upon exposure to the same pathogen or cross-reactive antigens, these memory cells are ready to fight again with greater speed and more efficiency (2). In some people, pre-existing memory cells generated against, for example, common cold coronaviruses, can cross-recognize the SARS-CoV-2 virus because of cross-reactive antigen binding between SARS-CoV-1 and SARS-CoV-2 (3, 4). This was shown by the generation of memory T cell lines that recognized many fragments from spike and non-spike regions of SARS-CoV-2, which were then tested for epitope similarity against a peptide pool of other coronaviruses. It was found that memory T cells made against SARS-CoV-2 cross-reacted with 57% of “common cold” coronavirus fragments (5, 6).

Furthermore, after the performance of antigen-specific T-cell studies, it was reported that 20-50% of individuals unexposed to SARS-CoV-2 had significant T-cell reactivity to various SARS-CoV-2 peptide sequences (2, 3, 7–10). This may be an answer to why, after contracting COVID-19, some people present only mild or moderate symptoms, but others get severely ill (11–13). The memory T cells generated against common cold coronaviruses may be responsible for this extensive heterogeneity in the human immune response to SARS-CoV-2 and its contribution to herd immunity (6, 13, 14).

All of this indicates that, immunologically, humans are not naïve, and that when they encounter new infections, the host immune system will activate its memory B and T cells, allowing quicker immune responses to a multitude of antigens, resulting in the production of both protective and cross-reactive antibodies (1, 2). In this context, we refer to the potential cross-reactivity to SARS-CoV-2 from common human pathogens and vaccines (15). This is based on the hypothesis that children may already have some degree of protection against SARS-CoV-2 due to the presence of cross-reactive immunity induced through their vaccinations with different bacterial and viral antigens (16–18). Because the immunity elicited by vaccines declines with aging, the adult population becomes more susceptible to COVID-19 (15). Of course, this possible cross-protection resulting from cross-reactive immunity most likely would not give the same degree of protection as specific SARS-CoV-2 vaccines.

Based on the hypothesis that the elderly are more prone to SARS-CoV-2 infection and children are largely spared due to pediatric vaccinations, a systemic search for peptide matches in 18 viruses and 7 bacteria with SARS-CoV-2 was conducted to identify potential cross-reactive epitopes by Reche in 2020 (15). While other researchers found that common herpesviruses were poor sources of cross-reactivity, Reche found that the combination of diphtheria, tetanus and pertussis (DTaP) in the DTaP vaccine proved to be a significant cause of cross-reactive immunity to SARS-CoV-2 (15). Comparing the amino acid sequences of overlapping 15-mer peptides from some of these pathogens with 10 SARS-CoV-2 residues, he reported as low as 1 epitope (polio virus) to 3,807 epitopes (*Bacillus Calmette-Guerin* or BCG) that cross-reacted with SARS-CoV-2 spike protein. Measles had 8 cross-reactive epitopes, HSV 1&2 had 77, Epstein-Barr virus 94, cytomegalovirus 169, diphtheria 340, tetanus 601, and *Bordetella pertussis* 3,359 (15). Based on these and other findings, Reche concluded that cross-reactive immunity elicited by DT antigens in combination with DTaP vaccines is likely responsible for keeping children safe from worldwide infection with SARS-CoV-2 (15).

In search of possible cross-reactivity between SARS-CoV-2 and human autoantigens, in our own earlier studies we first reacted animal and then human monoclonal antibodies (19, 20) made against SARS-CoV-2 spike protein and nucleoproteins with more than 55 human tissue antigens, and found that the SARS-CoV-2 antibodies had moderate to strong reactions with more than 20 of the human tissue antigens. We concluded that a potential risk for autoimmunity may come from cross-reactivity between SARS-CoV-2 antigens and our own tissue antigens (19, 20).

Furthermore, in additional studies we investigated antigenic mimicry between dietary proteins and human autoantigens not only by epitope sharing but also through the interaction of food-specific antibodies with human tissue antigens and vice-versa (21–24). We found that an extensive number of food antigens reacted with tissue-specific antibodies, and many food antibodies such as lectins and agglutinins reacted with numerous tissue antigens (25, 26). We also showed significant immunological cross-reactivity between different viruses and other pathogens (27). Observing this interaction between polyclonal and monoclonal antibodies made against food antigens and pathogens with various tissue antigens led us to hypothesize the following:

1. SARS-CoV-2 antibody may cross-react with common viral and bacterial antigens, including some which were not examined in the 2020 study by Reche (15).
2. SARS-CoV-2 proteins may share cross-reactive epitopes with many food antigens that have not previously been studied.
3. The production of cross-reactive antibodies against viral, bacterial and food antibodies may be responsible for extensive heterogeneity in their response to SARS-CoV-2 in different countries in the world.

To identify possible cross-reactive immunity to SARS-CoV-2, we reacted SARS-CoV-2 monoclonal antibodies with 14 different viral and bacterial antigens, and 180 different food antigens and peptides. We then reacted human sera with very low levels of antibodies to selected pathogens and food antigens versus sera with very high levels of these same antigens, for comparative purposes, with SARS-CoV-2 antigens. Finally, we conducted systematic searches for sequences shared by SARS-CoV-2, and pathogens and food antigens with which SARS-CoV-2 antibodies had reacted.

## Methods

### Pathogens and vaccines

*E. coli* and Salmonella lipopolysaccharides (LPS), and *Enterococcus faecalis* were purchased from Sigma Aldrich (St. Louis, MO USA).

ELISA microwell plates coated with *B. burgdorferi*, EBV-VCA, EBV-EAD, EBV-EBNA, CMV, VZV and measles antigens were purchased from Trinity Biotech (Jamestown, NY USA).

HHV-6 A and B were purchased from Bio-Synthesis (Lewisville, TX, USA).

ELISA well plates coated with HSV 1 + 2 antigens were obtained from Gold Standard Diagnostics (Davis, CA USA).

*H. influenza*, BCG and DTaP vaccines were purchased from the local pharmacy.

### SARS-CoV-2 antibodies and antigens

Human IgG_1_ monoclonal antibody made against SARS-CoV-2 spike protein S1 domain (CR3022) Catalog #NBP3-11813 was purchased from Novus Biologicals (Centennial, CO USA). This antibody binds specifically to amino acids 318-510 in the S (spike) domain of the SARS-CoV spike protein as well as the SARS-CoV-2 (COVID-19) spike protein

Human IgG_1_ monoclonal antibody made against SARS-CoV-2 nucleoprotein (CR3009) SKU: MAB12434 was obtained from The Native Antigen Company (Kidlington, Oxfordshire, UK). This antibody recognizes and binds the non-linear/conformational epitope of the N protein of SARS-CoV and SARS-CoV-2.

Recombinant SARS-CoV-2 spike protein S1 subunit and recombinant SARS-CoV-2 nucleocapsid protein were purchased from RayBiotech (Atlanta, GA USA).

### Food proteins and peptides

For the preparation of the food antigens, food products were purchased from a Whole Foods supermarket. Food proteins undergo structural epitope transformation when the food is cooked, so, when necessary, foods underwent preparation so that the resulting food proteins would accurately represent the raw and cooked foods of typical human diets. Using a process similar to the one used in our earlier study (28), a total of 180 different foods representing different meats, seafoods, vegetables, fruits, grains, nuts, seeds, beans, spices, gums and more were prepared.

Lectins and agglutinins such as wheat germ agglutinin (WGA), soybean agglutinin, phytohemagglutinin, peanut agglutinin and concanavalin A were purchased from Sigma Aldrich (St. Louis, MO USA).

Gliadin peptides were synthesized by Bio-Synthesis (Lewisville, TX USA).

Food products were ground at 4°C in either 70% ethanol, or coco buffer containing 0.55 M of NaHCO_3_, 1% NaCl pH 8.5. Each food item was left on the stirrer at 25°C for 4 h. The food processor was decontaminated after each food was stirred. The mixture was centrifuged for 15 minutes at 2000 g, after which the top layer containing oil bodies was discarded. To ensure that all small molecules were removed, each solvent’s liquid phase was dialyzed against a buffer of 0.01 M phosphate buffered saline (PBS) using dialysis bags with a cutoff of 6000 DA for 72 h, with the buffer changed every 24 h. The protein concentration was subsequently measured using a kit obtained from Biorad (Hercules, CA USA). The complete list of the 180 foods can be found in **Supplementary Material**.

### Antibodies against food proteins and peptides

Rabbit anti-gluten was purchased from Sigma Aldrich (St. Louis, MO USA).

Rabbit anti-phytohemagglutinin was obtained from Abcam (Fremont, CA USA).

Rabbit anti-WGA, anti-soybean agglutinin, anti-wheat, anti-a-gliadin, anti-egg, anti-corn, anti-peanut agglutinin and others were prepared by Bio-Synthesis (Lewisville, TX USA).

### Reaction of anti-SARS-CoV-2 polyclonal and monoclonal antibodies with different pathogens, vaccine antigens, and food proteins and peptides

Commercially available microwell plates coated with different bacterial and viral antigens including BCG vaccine, DTaP vaccine, SARS-CoV-2 spike protein and nucleoprotein were prepared at an optimal concentration of 1-3 mg/mL. After dilution at 1:100 in 0.01 M PBS, pH 7.4, 100 microliters containing 1-3 mg of these antigens were added to a series of 96-well microtiter plates.

Food antigens were prepared at a concentration of 1 mg/mL. In coating the ELISA plate, we determined the optimal concentration of each food antigen by examining the concentration of antigens that gave the most reproducible results in quadruplicate. Consequently, we diluted the stock solution from 1:25 to 1:100 in 0.1 M carbonate-bicarbonate buffer (pH 9.5). One hundred microliters were added to each well of the polystyrene flat-bottom ELISA plate. All plates were kept for 6 h at room temperature (RT) and 18 h at 4°C. Plates were washed 4 times using an ELISA washer, and 200 microliters of 2% BSA were added to each well and incubated for 24 h at 4°C in order to block the non-specific binding of the antibody to the antigen-coated wells. To examine the binding of SARS-CoV-2 antibodies to each one of these antigens, 100 microliters of human anti-spike protein and human anti-nucleoprotein at optimal dilutions of 1:100-1:200, and rabbit anti-envelope and rabbit anti-membrane proteins at a dilution of 1:100 were each added to quadruplicate wells of microtiter plates coated with various antigens. After 1 h of incubation and washing, an optimal dilution of alkaline-phosphatase-labeled anti-human or anti-rabbit IgG was added to the appropriate sets of plates, which were then incubated again for 1 h at RT. To remove the unbound antibodies, plates were washed 5 times, and 100 microliters of substrate para-nitrophenylphosphate were added. After 45 mins, color development was measured using an ELISA reader at 405 nm. We calculated the means of the respective quadruplicate wells and used them in the graphs.

We calculated the percentage of each antibody’s tissue reaction according to the following formula:


$$\text{\% of reaction with the antibody}=\frac{\text{OD of tissue reactivity–OD of background}}{OD of SARS-COV-2 reactivity–OD of background}\times 100$$

In order to determine the specificity of human monoclonal and rabbit polyclonal antibodies in binding to pathogens, vaccines and food antigens, these antibodies were replaced with the same dilution of human serum from a healthy subject or with non-immunized rabbit serum and added to quadruplicate wells. Furthermore, the antibodies and other reagents were added to 4 wells coated with 2% HSA and BSA alone and used as negative controls. After completion of all ELISA steps, the ODs of these control wells were measured.

### Binding of SARS-CoV-2 antibodies to serially diluted spike proteins, nucleoproteins, vaccine and food antigens

For the demonstration of the specificity of SARS-CoV-2 antibody binding to SARS-CoV-2 antigens, bacterial, viral, vaccine and food antigens were prepared at a concentration of 1 mg/mL. Each antigen was then diluted 1:200, 1:400, and 1:800, after which 100 microliters of each antigen was added to different rows of microtiter plates. This way, each row was coated with no antigen (blank), or with final amounts of 500, 250 and 125 nanograms of each antigen: *E. faecalis*, HSV 1 + 2, EBV EAD, DTaP, α+β casein, gliadin peptide, and peanut proteins. These antigens were chosen because they showed from low to strong reactivity with either SARS-CoV-2 spike and nucleoprotein antibodies. After the completion of the antigen-coating steps, SARS-CoV-2 monoclonal antibodies at a dilution of 1:100 was added to the wells coated with different concentrations of antigens. After completion of the other ELISA steps, the ODs were recorded at 405 nM.

### Reaction of food-specific antibodies with SARS-CoV-2 spike proteins and nucleoproteins

Different wells of ELISA plates were coated with 0.5 micrograms of either spike protein or nucleoprotein that had been dissolved in 100 ml of 0.01 M PBS pH 7.4 and were then kept for 8 h at RT followed by 16 h at 4°C. Plates were washed 4 times with 0.01 M PBS containing 0.05% Tween 20. After washing, 200 ml of 2% BSA was added and incubated again for 8 h at RT, and 16 h at 4°C to block the uncoated surfaces. Following removal of the BSA and washing, the plates were ready for antibody reactivity. 100 microliters of serum diluent were added to the first 4 wells of a microtiter plate coated with spike protein or nucleoprotein. 100 microliters of unimmunized rabbit serum diluted 1:100 was added to the second set of 4 wells, and 100 microliters of rabbit anti-spike or anti-nucleoprotein antibody was added to a third set of 4 wells. The second and third sets of wells, 4 in each set, 8 in total, served as negative and positive controls. 100 microliters of rabbit serum with very high titers of IgG antibody to wheat, α-gliadin, WGA, milk, α+β casein, soy, soy agglutinin, peanut agglutinin, phytohemagglutinin, egg, and corn antibodies diluted 1:100. After 60 mins of incubation and washing, 100 microliters of goat anti-rabbit IgG labeled with alkaline phosphatase at a dilution of 1:200 was added. Color development and optical densities were recorded at 405 nM after completion of ELISA steps.

### Reaction of human sera containing low or high levels of antibodies to viral and food antigens with SARS-CoV-2 spike protein

Using ELISA methodology, we screened 200 sera for the presence or absence of IgG antibodies against EBV EAD, HSV 1 + 2, HHV-6, peanut proteins, wheat, gliadin peptide, gluteomorphin + dynorphin, milk, α + β casein, and pineapple bromelain. We then selected 24 sera with very low levels and 24 sera with very high levels of antibodies against each of these viral or food antigens-coated plates. After dilution of each serum 1:50, and the completion of ELISA steps, the ODs were compared.

### Reaction of human sera with low or high levels of SARS-CoV-2 antibody with other viral, food, and vaccine antigens

Using SARS-CoV-2 Zeus ELISA, we screened many sera and selected 24 with non-detectable and 24 with high levels of IgG antibody to SARS-CoV-2 spike protein and nucleoprotein. We then applied the selected sera at a dilution of 1:20 to ELISA plates coated with EBV EA, CMV, HSV 1 + 2, HHV-6, DTaP and BCG vaccines, peanut butter, wheat, gliadin peptide, gluteomorphin + dynorphin, milk, α+β casein, and pineapple bromelain. The selection of these vaccines, viral and food antigens was based on their reactivity to SARS-CoV-2 monoclonal antibody. After completion of all the ELISA steps, the ODs were recorded.

### Amino acid sequence similarity between SARS-CoV-2 spike protein, nucleoprotein and other viruses and food antigens

We used the NIH/US National Library of Medicine’s BLAST (Basic Local Alignment Search Tool) sequence matching program to study the degrees of possible mimicry or amino acid (AA) sequence homology shared by SARS-CoV-2 spike protein and nucleoprotein with EBV EA, EBV VCA, EBV EBNA, CMV, HSV-1, HSV-2, HHV-6, peanut ARA H2 allergen, peanut agglutinin, wheat gliadin, glutenin, wheat germ agglutinin, casein, lentil lectin, pineapple bromelain and rice endochitinase.

### Statistical analysis

Statistical analysis was performed by comparing the ODs obtained for the reactive tissue antigens with the mean OD of non-reactive tissue antigens + 3SD using STATA 14.2 software. Independent t-tests were performed to evaluate mean differences of optical densities between controls and antigens. A Bonferroni adjustment was conducted to account for type 1 errors with multiple comparisons and alpha was set to< 0.001.

## Results

### Reaction of human SARS-CoV-2 monoclonal antibodies with different vaccine, bacterial and viral antigens

We used human monoclonal antibodies made against SARS-CoV-2 spike proteins and nucleoproteins to measure the degree of immune reactivity of these antibodies with 15 different bacterial and viral antigens and 2 different vaccines, Bacillus Calmette-Guérin (BCG) and Diphtheria, Tetanus and Pertussis (DTaP). An earlier study showed that these 2 vaccines had a high degree of amino acid sequence similarity with SARS-CoV-2 (18). As expected, in comparison to the blank OD of 0.1 or less, the reaction of spike protein and nucleoprotein antibodies with recombinant spike proteins and nucleoproteins was greater than 3.5, which is very close to the maximum detection limit of the assay.

**Figure 1:**
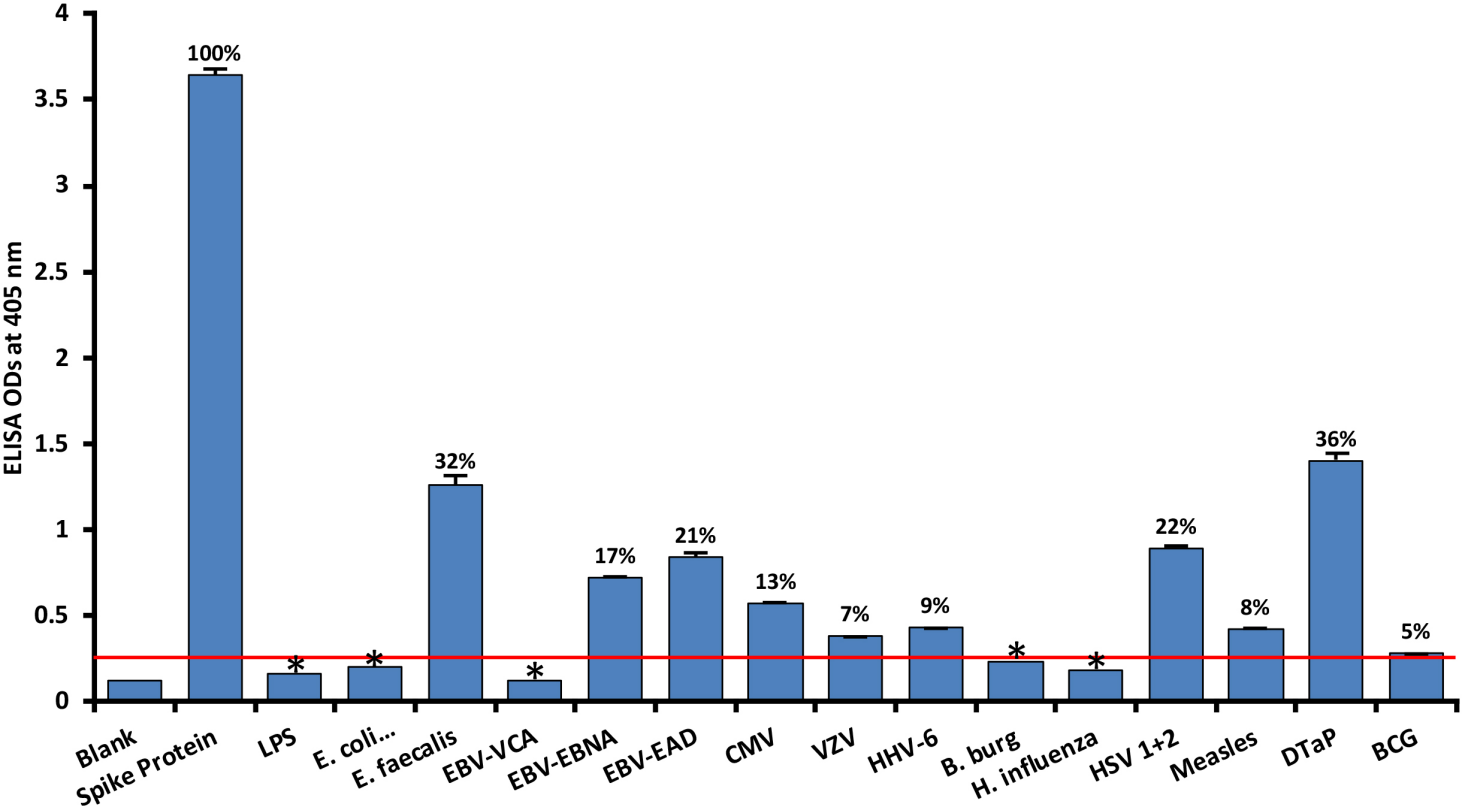
Reaction of human SARS-CoV-2 spike protein monoclonal antibody with spike protein and different bacterial and viral antigens, including DTaP and BCG vaccines. At 3SD above the OD of background or 0.27, significant reaction between spike protein antibody and bacterial and viral antigens was observed. Each determination of antigen-antibody reaction was performed in quadruplicate. The standard deviations (SDs) for all the reactions were less than 0.1, and are shown as error bars. In these experiments, when monoclonal anti-SARS-CoV-2 antibodies were replaced with sera from healthy subjects, non-significant reactions with a mean of 0.25 were observed from the sera with spike protein. Percentages of significant reactivity are shown. DTaP is shown to be the most reactive. * = Antigens whose reactions were under the cutoff and/or close to the blank.

As shown in **Figure 1**, with spike protein reactivity as 100%, significant immune reactivity was observed between spike protein monoclonal antibody and DTaP vaccine (OD 1.4, or 36%), *E. faecalis* (OD 1.3 or 32%), and HSV 1 + 2 (OD 0.9, or 22%), but not with BCG vaccine. The immune reactivity was lower with CMV and most EBV antigens, ranging from 0.58 (13%) – 0.84 (21%) ODs. The ODs for VZV, measles and HHV-6 ranged from 0.38 (7%) – 0.43 (9%). Finally, for antigens such as LPS, *E. coli*+Salmonella CDT peptides, EBV VCA, and *Borrelia burgdorferi*, the ODs were very close to 3SD above the background OD of the controls, or OD< 0.27. The percentages of significant reactivity between SARS-CoV-2 spike antibodies and different bacterial and viral antigens are also shown in **Figure 1**. Overall, DTaP, *E. faecalis*, HSV 1 + 2, EBV-EAD, EBV-EBNA, CMV, HHV-6, measles, and VZV had significant *p* values (*p*< 0.001), while the other antigens were insignificant.

**Figure 2:**
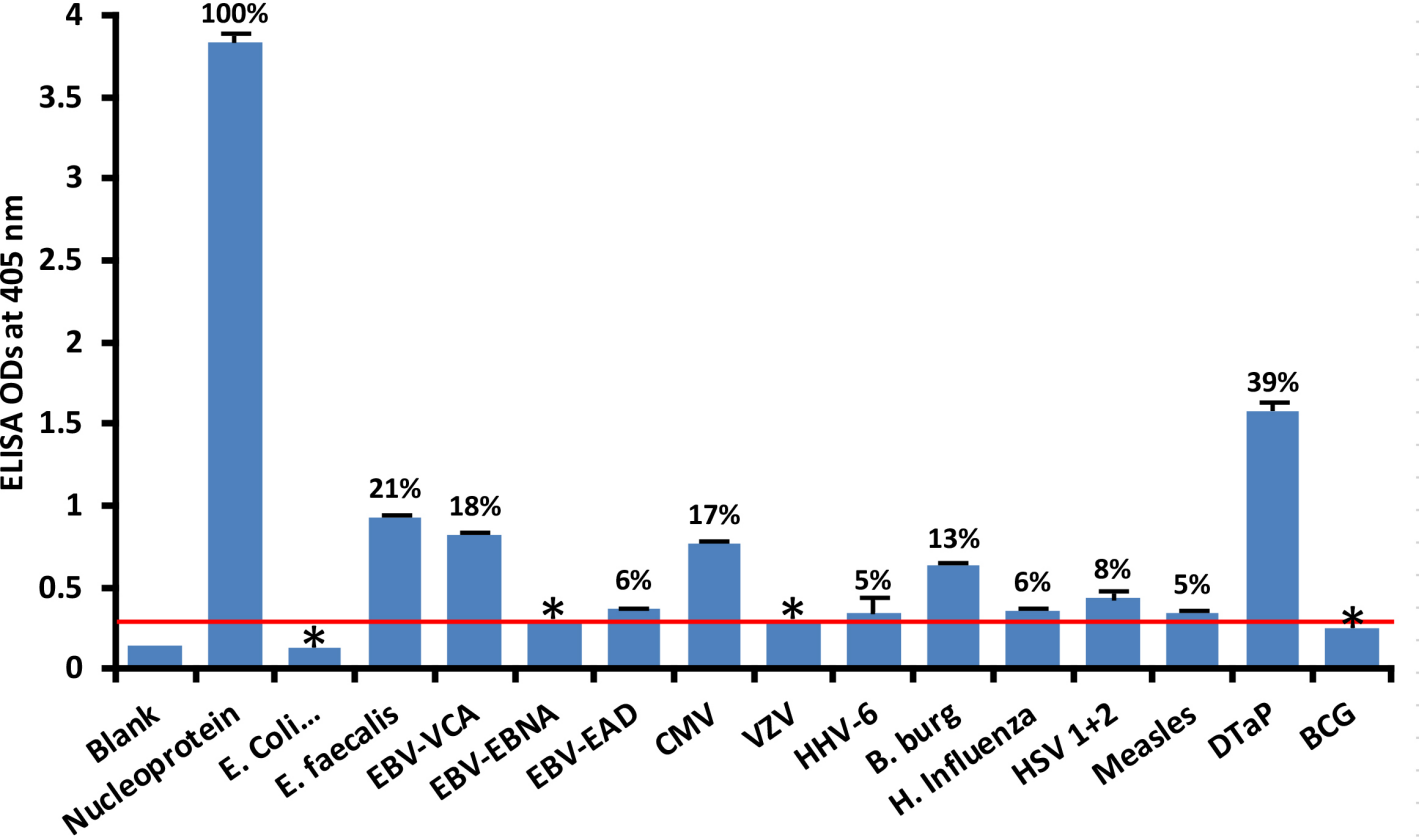
Reaction of human SARS-CoV-2 nucleoprotein monoclonal antibody with nucleoprotein and different bacterial and viral antigens, including DTaP and BCG vaccines. At 3SD above the OD of background or 0.3, significant reaction between spike protein antibody and bacterial and viral antigens was observed. Each determination of antigen-antibody reaction was performed in quadruplicate. The SDs for all the reactions were less than 0.12, and are shown as error bars. In these experiments, when monoclonal anti-SARS-CoV-2 antibodies were replaced with sera from healthy subjects, non-significant reactions with a mean of 0.29 were observed from the sera with nucleoprotein. The ODs of these reactions were lower than 0.3. Percentages of significant reactivity are also shown. DTaP is shown to be the most reactive. * = Antigens whose reactions were under the cutoff and/or close to the blank.

At 3SD above the background OD of the controls (0.3), the human monoclonal antibody to nucleoprotein had weak to strong reactions with 10 out of the 14 bacterial and viral antigens. As shown in **Figure 2**, with nucleoprotein reactivity as 100%, the strongest immune reactivity was with DTaP (OD 1.58 or 39%), *E. faecalis* (OD 0.93 or 21%), EBV-VCA (OD 0.82 or 18%), CMV (OD 0.77 or 17%) and *Borrelia burgdorferi* (OD 0.63 or 13%), all of which had *p* values< 0.001. For *E. coli* + Salmonella CDT peptides, EBV-EBNA, EBV-EAD, VZV, HHV-6, *H. influenza*, HSV 1 + 2, measles, and BCG, the ODs were below the cutoff or very close to the blank, and their *p* values were insignificant. The percentages of significant reactivity between SARS-CoV-2 nucleoprotein antibodies and different bacterial and viral antigens are also shown in **Figure 2**.

### Reaction of human SARS-CoV-2 monoclonal antibodies with different food antigens and peptides

Similar to what we did with pathogen and vaccine antigens, we reacted monoclonal antibody made against SARS-CoV-2 spike proteins and nucleoproteins with 180 different commonly consumed food proteins and peptides.

**Figure 3:**
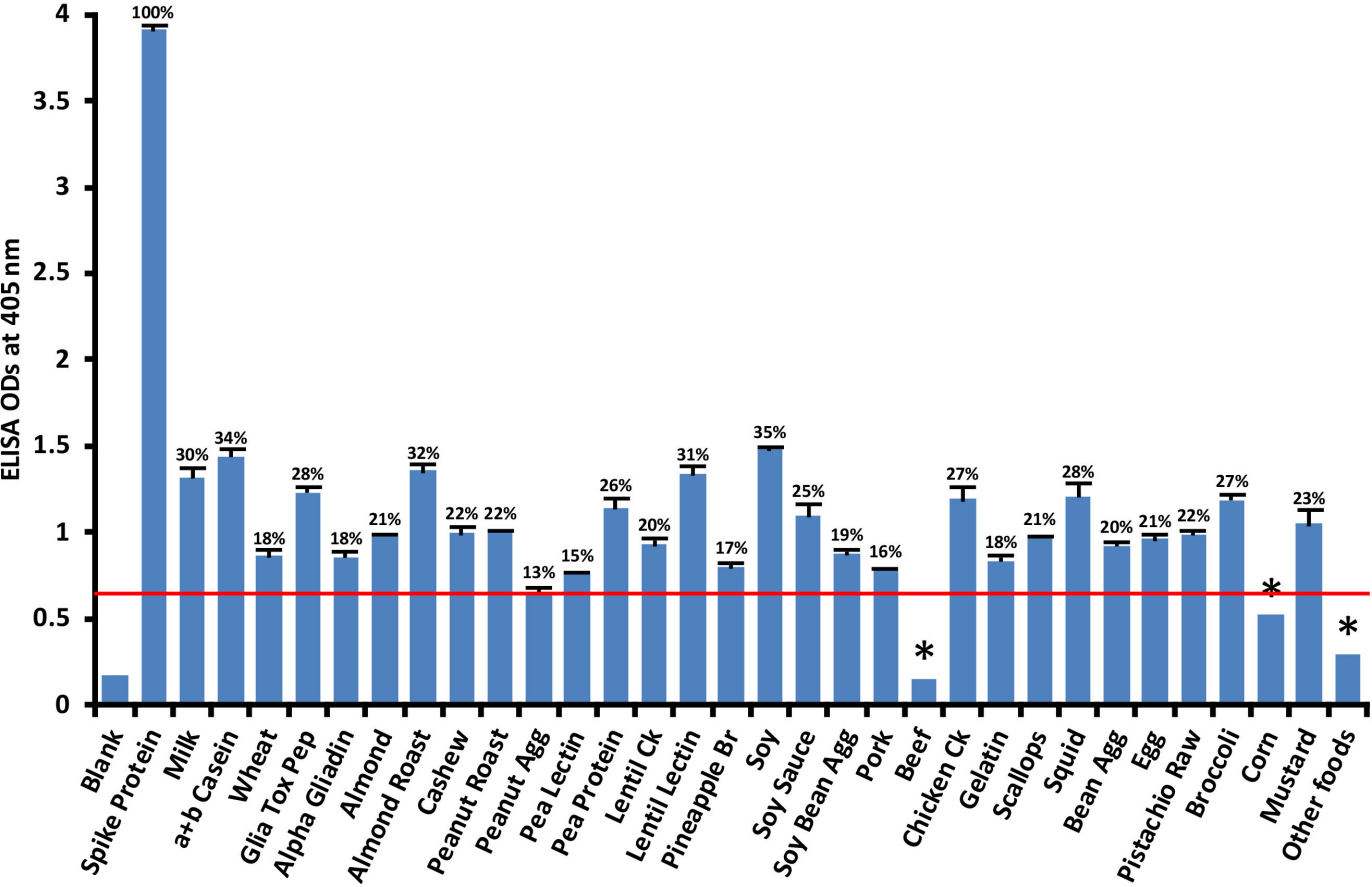
Reaction of human SARS-CoV-2 spike protein monoclonal antibody with various food antigens. At 3SD above the OD of antibody reaction with non-reactive foods or OD of 0.56, the spike protein antibody reacted significantly with 28 out of 180 tested food proteins and peptides. These reactions of SARS-CoV-2 spike protein antibody with different food antigens was obtained from quadruplicate testing. The SDs for all the reactions were less than 0.1, and are shown as error bars. * = Antigens whose reactions were under the cutoff and/or close to the blank. Glia Tox Pep, Gliadin Toxic Peptide; Peanut Agg, Peanut Agglutinin; Soy Bean Agg, Soy Bean Agglutinin; Bean Agg, Bean Agglutinin; Pineapple Br, Pineapple Bromelain; Ck, Cooked.

Anti-SARS-CoV-2 spike protein antibody had a significant reaction with 28 out of 180 food antigens. Reactions with beef and corn were weaker. The reactivity between SARS-CoV-2 monoclonal antibody and different foods was considered positive only if the obtained ODs were higher than the reagent controls and the mean ODs of the other foods + 3SD. The cutoff OD for spike protein antibody reaction with various foods was determined to be 0.56. **Figure 3** shows that the most significant reactions (*p*< 0.001) of spike protein antibody were with soy (35%), α+β casein (34%), roasted almond (32%), lentil lectin (31%), milk (30%), gliadin toxic peptide (28%), squid (28%), cooked chicken (27%), broccoli (27%), and pea protein (26%).

**Figure 4:**
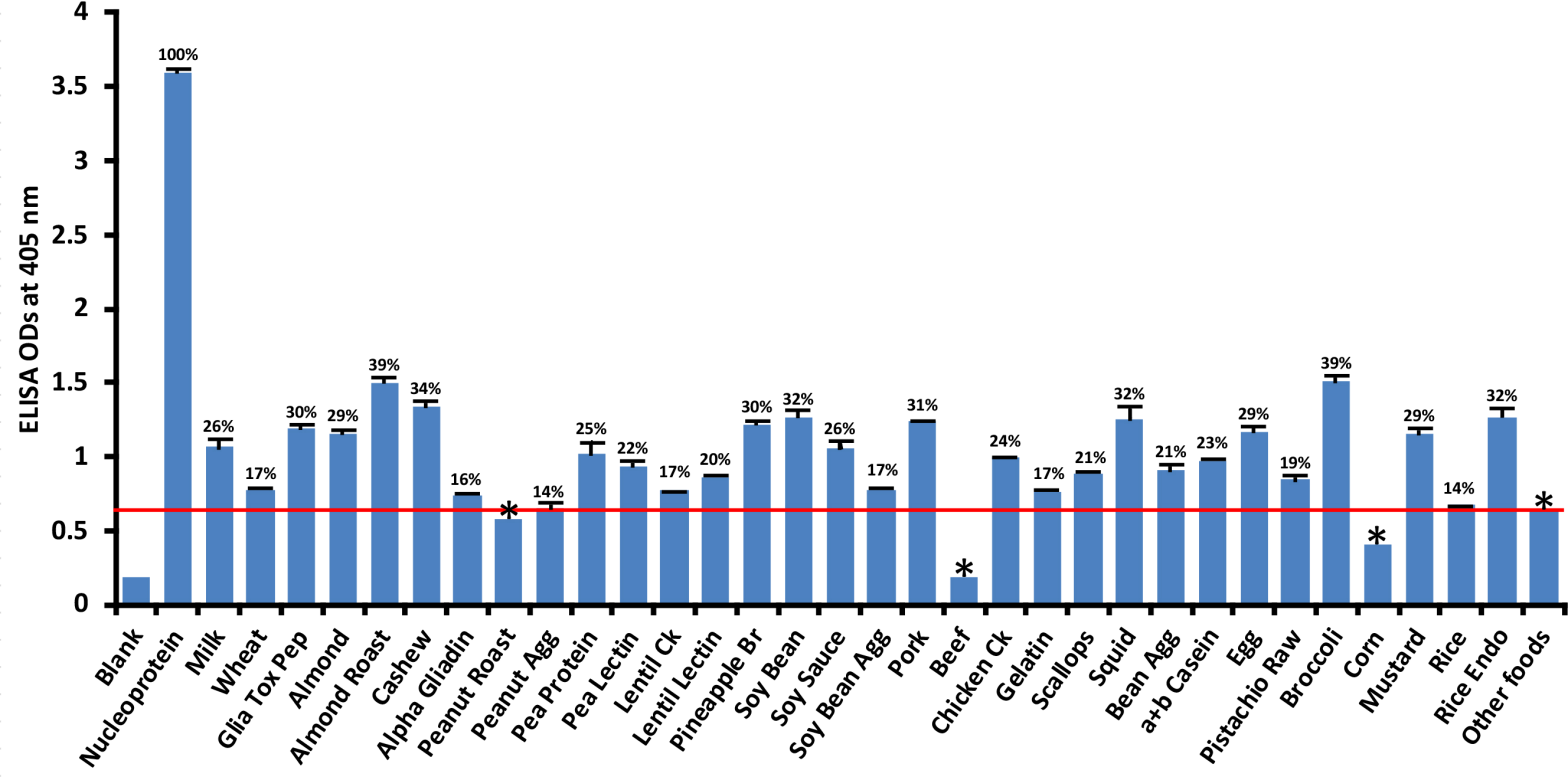
Reaction of human SARS-CoV-2 nucleoprotein monoclonal antibody with various food antigens. At 3SD above the OD of antibody reaction with non-reactive foods or OD of 0.64, the nucleoprotein antibody reacted significantly with 26 out of 180 tested food proteins and peptides. The data was obtained from quadruplicate testing. The SDs for all the reactions were less than 0.1, and are shown as error bars. * = Antigens whose reactions were under the cutoff and/or close to the blank. Glia Tox Pep, Gliadin Toxic Peptide; Peanut Agg, Peanut Agglutinin; Soy Bean Agg, Soy Bean Agglutinin; Bean Agg, Bean Agglutinin; Pineapple Br, Pineapple Bromelain; Rice Endo, Rice Endochitinase; Ck, Cooked.

Compared to its reaction with SARS-CoV-2 nucleoprotein (100%), the application of SARS-CoV-2 nucleoprotein antibody to 180 food antigens resulted in the strongest reactions with the following foods: broccoli (39%), roasted almond (39%), cashew (34%), soy bean (32%), squid (32%), rice endochitinase (32%), pork (31%), pineapple bromelain (30%), and gliadin toxic peptide (30%). The cutoff OD for nucleoprotein was 0.64. The reactions with an additional 24 foods were not as strong; those still above the cutoff but weaker ranged from peanut agglutinin (14%) to egg (29%) while roasted peanut, beef and corn were below the cutoff (**Figure 4**). Overall, the difference in reactivity of SARS-CoV-2 monoclonal antibody with 22 of the reactive foods was very significant at *p*< 0.001, significant with 4 foods at *p* = 0.001, and insignificant for the rest.

### Serial dilutions of human SARS-CoV-2 spike and nucleoprotein antibodies with the same concentration of vaccines and food antigens

**Figure 5:**
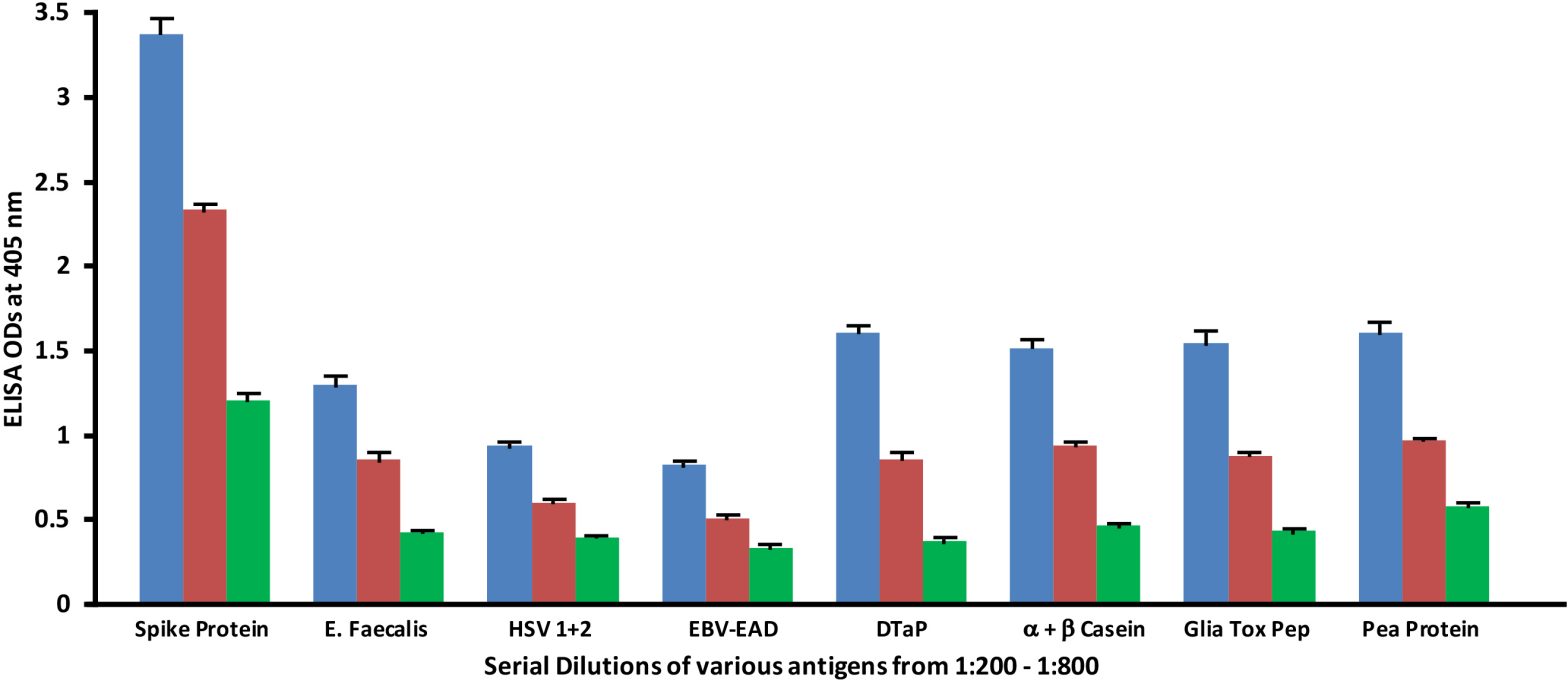
Reaction of human SARS-CoV-2 spike protein monoclonal antibody with different bacterial, viral and food antigens at dilutions of 1:200 
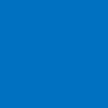
, 1:400 
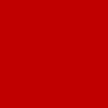
, and 1:800 
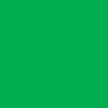
. Note that in proportion to the dilution of the antigens, a significant decline in antibody-antigen reaction is observed. Each determination of antibody-antigen reaction was performed in triplicate. The SDs for all the reactions were less than 0.1, and are shown as error bars.

Monoclonal antibodies made against both spike protein and nucleoprotein were applied to spike protein and nucleoprotein, as well as bacterial, viral and food antigens that had been serially diluted at dilutions of 1:200, 1:400 and 1:800. These antigens were selected because they had reacted significantly with these antibodies in prior experiments. As shown in **Figure 5**, the reaction of the anti-SARS-CoV-2 spike protein antibodies with *E. faecalis*, HSV 1 + 2, EBV-EAD, DTaP vaccine, α+β casein, gliadin toxic peptide and pea protein decreased in proportion to an increase in the dilution.

**Figure 6:**
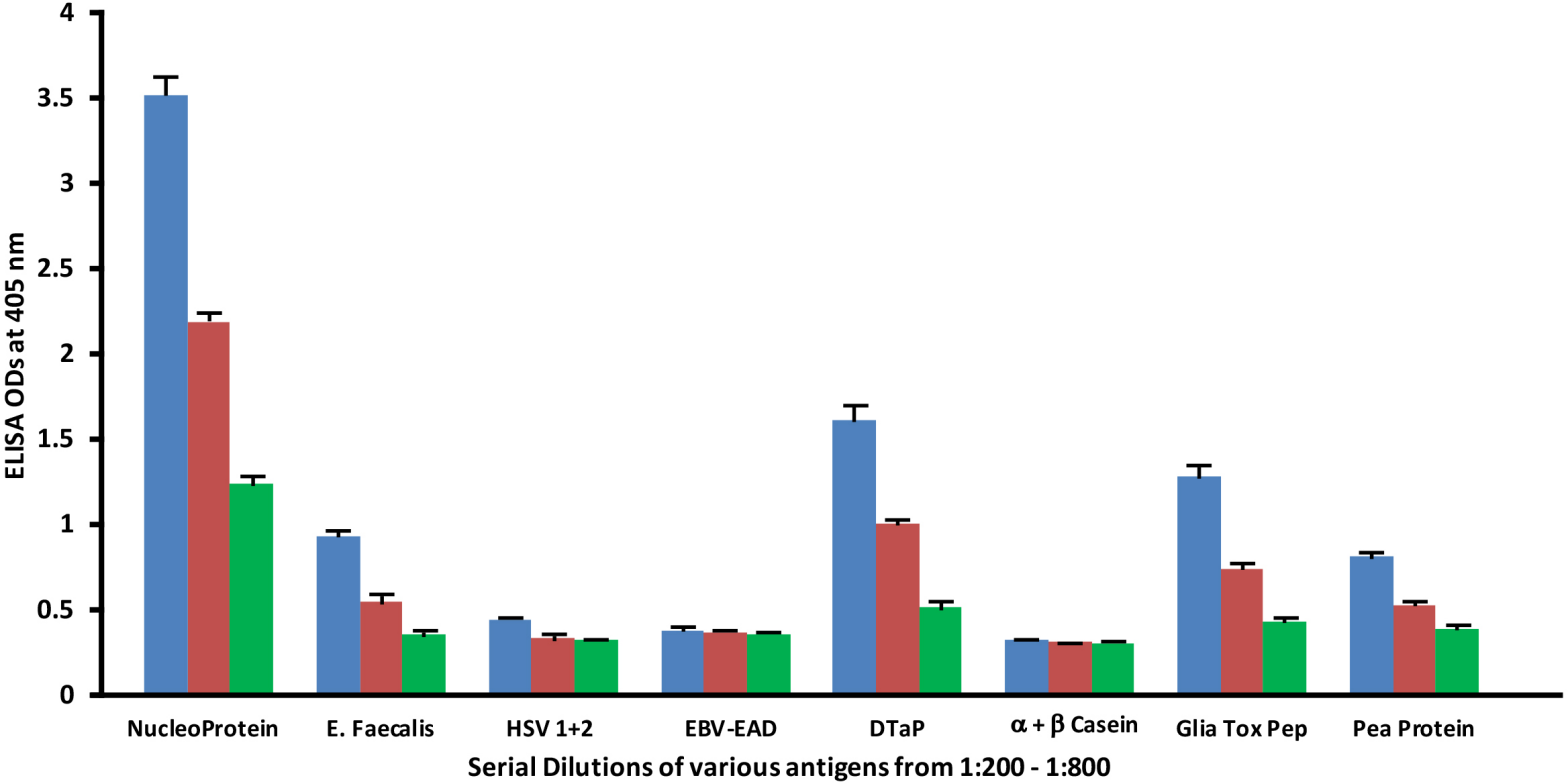
Reaction of human SARS-CoV-2 nucleoprotein monoclonal antibody with different bacterial, viral and food antigens at dilutions of 1:200 
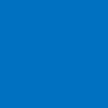
, 1:400 
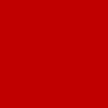
, and 1:800 
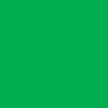
. Note that in proportion to the dilution of the antigens, a significant decline in antibody-antigen reaction is observed only in the antigens with ODs that are >0.5. Each determination of antibody-antigen reaction was performed in triplicate. The SDs for all the reactions were 0.1 or less, and are shown as error bars. .

Similar to spike protein antibody, the reaction of SARS-CoV-2 nucleoprotein antibody with bacterial, viral and food antigens decreased in proportion to an increase in the dilution (**Figure 6**). This corollary decline, however, is more pronounced and noticeable with antigens that reacted strongly with anti-nucleoprotein antibody, such as DTaP, gliadin toxic peptide, *E. faecalis*, and pea protein, while the decline with HSV 1 + 2, EBV-EAD and α+β casein, although present, are less obvious due to the closeness of their ODs to the background.

### Reaction of rabbit polyclonal affinity-purified food-specific antibodies with SARS-CoV-2 spike protein and nucleoprotein

**Figure 7:**
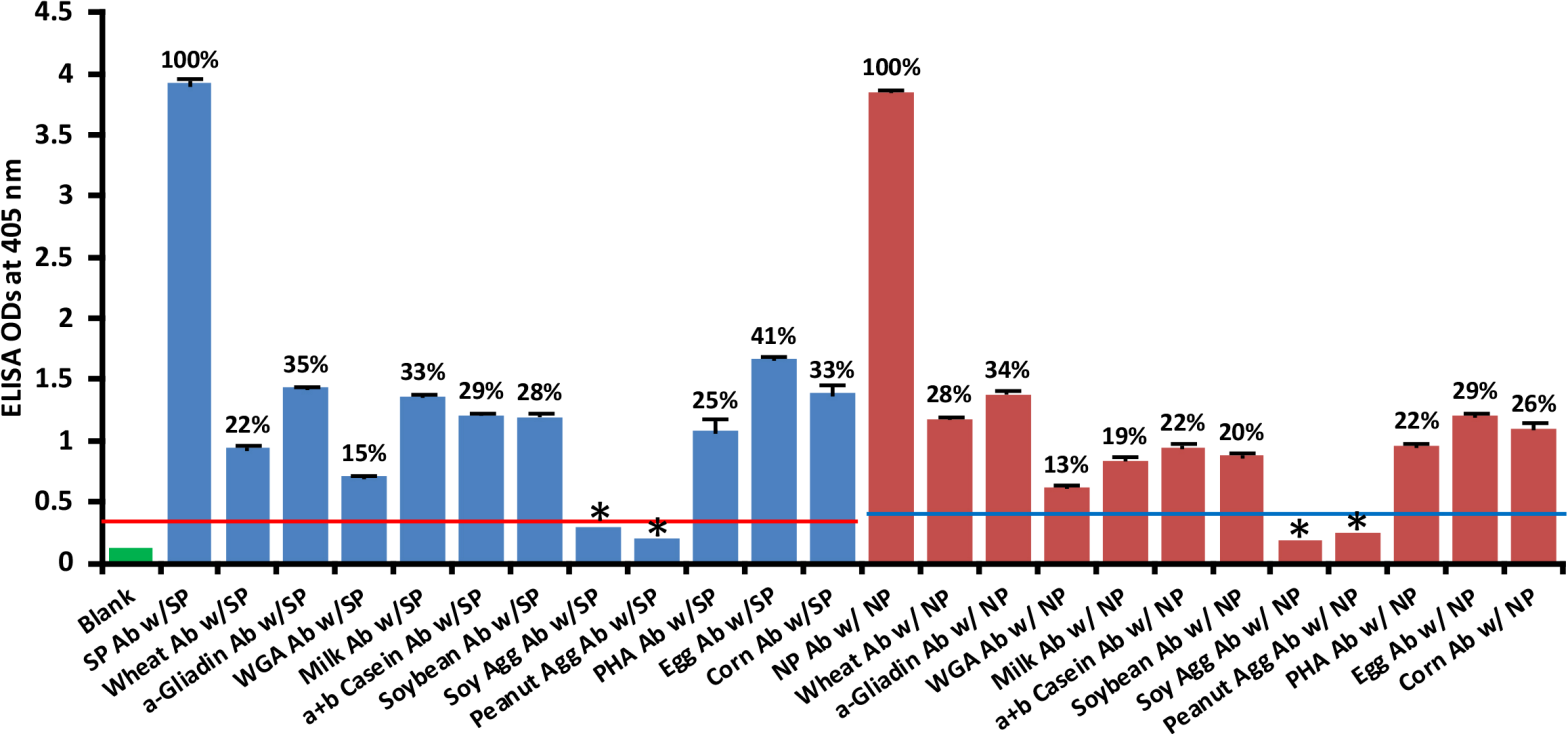
Reaction of affinity-purified rabbit polyclonal antibodies made against different food antigens with recombinant SARS-CoV-2 spike 
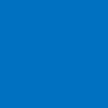
or nucleoprotein 
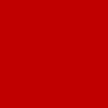
. Each determination of antigen-antibody reaction was performed in quadruplicate. The SDs for all the reactions were 0.1 or less, and are shown as error bars. * = Antigens whose reactions were under the cutoff and/or close to the blank. WGA, Wheat Germ Agglutinin; Peanut Agg, Peanut Agglutinin; Soy Agg, Soy Agglutinin; PHA, Phytohemagglutinin. Percentages of significant reactivity are also shown.

Using ELISA methodology, we reacted affinity-purified antibodies made against phytohemagglutinin (PHA), soy protein, soy agglutinin, peanut agglutinin, wheat, wheat germ agglutinin (WGA), α-gliadin-33 mer, milk, α+β casein, egg and corn by applying them to ELISA microwells coated with spike protein or nucleoprotein. We found that unimmunized rabbit serum diluted 1:100 did not react significantly with SARS-CoV-2 proteins. The ELISA indices for the unimmunized rabbit serum for all the reactions were within 3SD above the mean OD of control wells (0.36 – 0.39). The reactions and percentages of reactivity between SARS-CoV-2 antibodies and different food antigens are shown in **Figure 7**.

The data presented in **Figure 7** show that the following food antibodies had moderate to strong reactions with both SARS-CoV-2 spike proteins (SP) and nucleoproteins (NP): anti-wheat (SP 22%, NP 28%), anti-α-gliadin (SP 35%, NP 34%), anti-milk (SP 33%, NP 19%), anti-α+β casein (SP 29%, NP 22%), anti-soy (SP 28%, NP 20%), anti-PHA (SP 25%, NP 22%), anti-egg (SP 41%, NP 29%) and anti-corn (SP 33%, NP 26%). The reactions of WGA antibody with the SARS-CoV-2 proteins were low (SP 15%, NP 13%), and the reactions of anti-soy agglutinin and anti-peanut agglutinin with those proteins was comparable to reaction of unimmunized rabbit serum with SARS-CoV-2 spike and nucleoproteins. To facilitate the clarity of results, the percentages of reactivity are also shown in **Figure 7**.

### Reaction of human sera containing low or high levels of antibodies to different viral and food antigens with spike protein

**Figure 8:**
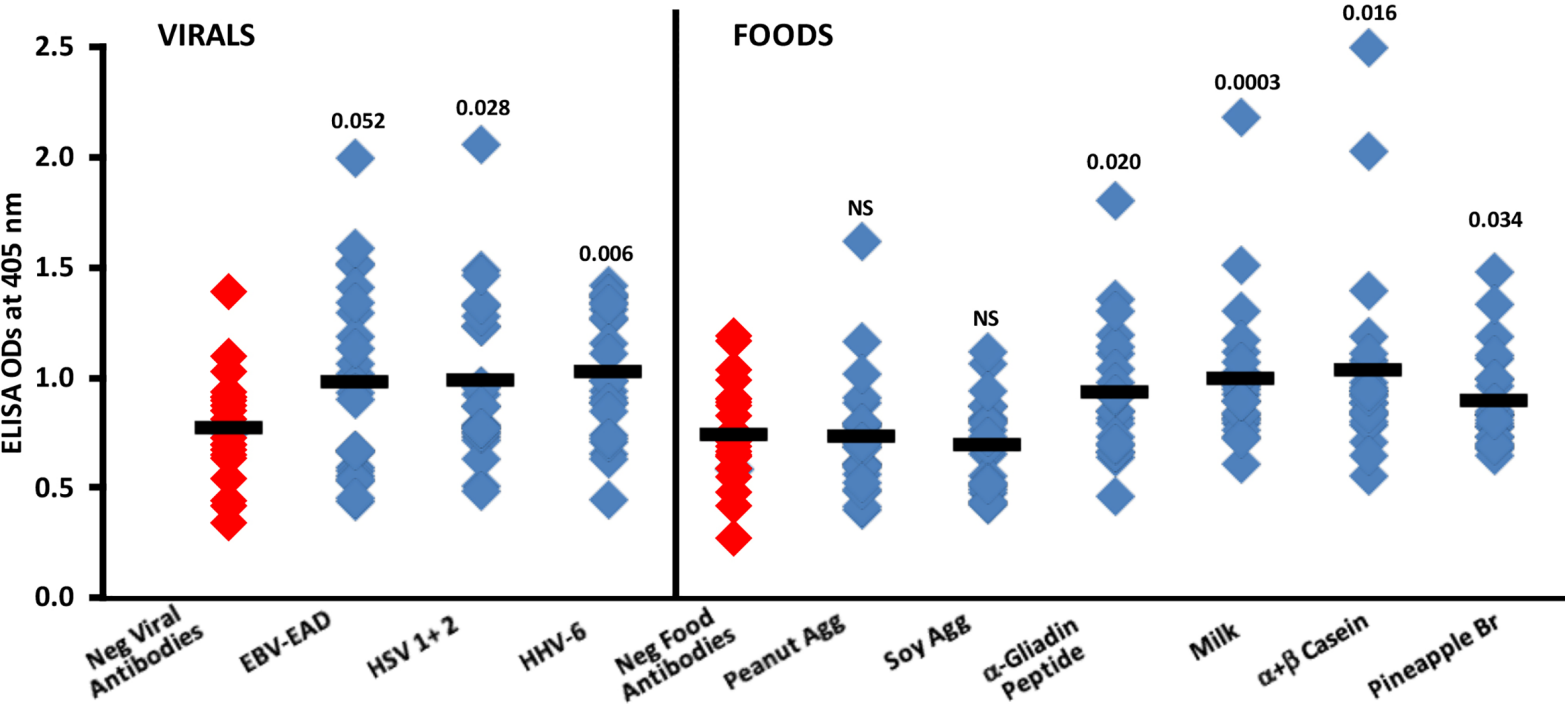
Reaction of human sera with low (Negative) or high levels of IgG antibody against EBV-EAD, HSV 1 + 2, HHV-6 and different food antigens with SARS-CoV-2 spike protein-coated plates. Black bars = means.

Summary results of the reaction of sera containing low or high levels of IgG antibodies against EBV-EAD, HSV 1 + 2 and HHV-6 with SARS-CoV-2 spike protein are shown in **Figure 8**. Compared to the reaction of 24 sera with low levels (negative) of IgG antibody against EBV-EAD, HSV 1 + 2 and HHV-6, the reaction of 24 sera containing high levels of antibodies against these viral antigens with SARS-CoV-2 spike protein resulted in higher ELISA ODs with *p* values of 0.052, 0.028 and 0.006 respectively for EBV-EAD, HSV 1 + 2, and HHV-6.

In **Figure 8**, the data shows that the reactions of human sera containing high levels of IgG antibody to peanut agglutinin and soy agglutinin with spike protein were comparable to those of sera containing low levels (negative) of antibody with non-significant *p*-values. However, most sera with high levels of IgG antibody against α-gliadin peptide, milk, α+β casein, and pineapple bromelain reacted strongly with SARS-CoV-2 spike protein, resulting respectively in *p* values of 0.020, 0.0003, 0.016, and 0.034.

### Amino acid sequence similarity between SARS-CoV-2 proteins and other viruses and food antigens

We used BLAST to find the degree of identity between SARS-CoV-2 proteins and other viruses and pathogens, including HSV-1, HSV-2, EBV, CMV, HHV-6, measles, VZV and *Borrelia burgdorferi*. SARS-CoV-2 proteins shared a significant number of peptides with each of these pathogens, as can be seen in **Table 1A** and **Table 1B**. These SARS-CoV-2 sequences shared 50-100% identity with different viruses. An almost similar number of peptide sequences with identity percentages ranging from 30 to 49% were also observed but are not shown in these tables. Similar to SARS-CoV-2 homology with other viruses and pathogens, we used BLAST to find a significant number of peptides from different foods that are consumed on a daily basis which shared 50-73% identity with SARS-CoV-2 sequences. These foods were peanuts, almonds, wheat, milk, rice, lentil and pineapple (see **Table 2A** and **Table 2B**). In both cases these subject sequences actually made a match with more than one section of the SARS-CoV-2 sequences; the multiple matches are indicated by asterixes in **Tables 1(A & B)** and **2(A & B)**.

**Table 1:** Potential cross-reactive epitopes between SARS-CoV-2 proteins and Herpesvirus antigens.


	SARS-CoV-2 antigen	SARS-CoV-2 sequence	Mapped start to end	Herpesvirus sequence	ID (%)
**HSV-1**	Chain R, Spike protein S1, SARS-CoV-2	LYFQGGSGDS	14-23	LY—DSGDS	60
	Chain A, Spike protein S1, SARS-CoV-2	VADYSVLYNS	56-65	VAGFLALYDS*	50
	Chain A, Spike protein S2, SARS-CoV-2	QLIRAAEIRAS	309-319	QLERVLETAAS*	55
	Chain A, Spike glycoprotein, SARS-CoV-2	LPDPSKPSKR	804-813	LPSVSLATKR*	50
	Chain A, Spike glycoprotein, SARS-CoV-2	VLFQGPGSGGLNDIFEAQ	1241-1258	VLFSGPSP–L—EAQ	50
	SARS-CoV-2 spike D614G variant, minus RBD, SARS-CoV-2	PGSGYIPEAP	1220-1229	PARGKYNGAP	50
**HSV-2**	Chain B Spike glycoprotein, SARS-CoV-2	AAYYVGYLQPRT	251-262	AA–IAYL–RT*	50
	Dissociated S1 domain of SARS-CoV-2 spike bound to ACE2 (non-uniform refinement), SARS-CoV-2	VFNATRFASV	372-381	VFFAASFAAI*	50
	Chain A, Spike protein S2, SARS-CoV-2	NDILSRLDKVEA	276-287	NDLISR-D–EA*	58
	Chain A, Spike glycoprotein, SARS-CoV-2	NDILSRLDPPEA	952-962	NDLISR-D–EA*	58
	Chain A, Spike glycoprotein, SARS-CoV-2	NDILSRLDKCEA	965-976	NDLISR-D–EA	58
	Chain A, Spike glycoprotein, SARS-CoV-2	PGDSS–SGWTAGA-AA	251-264	PTDSSILS—PGALAA*	53
	Chain A, Spike glycoprotein, SARS-CoV-2	DS–LSSTASAL	936-945	DSSILSP–GAL*	50
	Chain A, Spike glycoprotein, SARS-CoV-2	DS–LSSTPSAL	936-945	DSSILS–PGAL*	58
**EBV-VCA**	Chain A, Spike glycoprotein, SARS-CoV-2	LS-TFLSGLEVLF	1233-1244	LSLTF—–VLF	54
	Chain A, Spike glycoprotein, SARS-CoV-2	TM–SLGAENSV	670-679	TMAKSL–ENSV*	67
	Chain A, Spike glycoprotein, SARS-CoV-2	LG–VENS–VAYS	696-705	LGCTVEKGDHVAYS*	57
	Chain A, Spike glycoprotein, SARS-CoV-2	TTLDSKTQSLLI	108-119	TTVEKK–SLTI*	50
	Chain A, Spike protein S2, SARS-CoV-2	YICGDSTECSN	34-49	YICTVSNPISN	55
**EAD**	Chain A, Spike protein S1, SARS-CoV-2	ALDPLSETKC	280-289	ALAVLS–KC*	60
	Chain B Spike glycoprotein, SARS-CoV-2	RPSQAEFGTATM	82-93	RP—EFVKLTM	50
	Chain E, Spike protein S1, SARS-CoV-2	VK-GFNCYFPL	151-160	VKQAFN–PL	55
**EBV-EBNA**	SARS-CoV-2 spike D614G variant, minus RBD, SARS-CoV-2	GSPGSGYIPEAPRGDQ	1218-1233	GPPGIG–PEGPL-GQ*	56
	SARS-CoV-2 spike D614G variant, minus RBD, SARS-CoV-2	GSPGSGYIPEAPR	1218-1230	GSP-SG—–PR*	54
	Chain A, ORF3a protein, SARS-CoV-2	EPIYDEPTTTT—SV	261-273	EP—PTVTTQRQSV	50
**CMV**	Chain A, Spike glycoprotein, SARS-CoV-2	KCVNFN—FNGLTG	511-522	KC—NDKKFNG-TG*	53
	Chain A, Spike protein S2, SARS-CoV-2	IRAA–EIRASAN	311-321	IRQAHCNI–SAN*	54
	Chain D, Spike protein S2, SARS-CoV-2	LEEVAKKLEE	20-29	LKQVAQKLHE	60
	Chain A, Spike protein S1, SARS-CoV-2	TPINLVRDLPQGFSAL	195-210	TSIRLV-D—GFLAL*	56

**HHV-6**	Crystal Structure of NendoU (Uridylate-specific endoribonuclease, nsp15)	SHHHHHHSSG	4-13	SHHHHHHSSG	100
	Peptide-bound SARS-CoV-2 Nsp9 RNA-replicase	HHHHHHSAAL	3-12	HHHHHHSSGL	80
	Crystal structure of 2019-nCoV nucleocapsid N-terminal domain (NTD) protein	HHHHHH–GL	1-8	HHHHHHSSGL*	80
	Structure of the RNA-dependent RNA polymerase	QVLSEM–VM	652-659	EVL—MMDVM*	50
	SARS-CoV-2 Envelope Protein Transmembrane Domain: Pentameric Structure Determined by Solid-State NMR	LFLAFV—VF	12-19	LFIVFVLLYVF	55
	Cryo-EM structure of SARS-CoV-2 ORF3a	IVGVALLAVFQ	47-57	IVFV-LLYVFH	64
	Cryo-EM structure of SARS-CoV-2 Spike ectodomain	LTDEMIAQYT	852-861	LTDNRTIVYT*	50
	X-ray Crystallographic Structure of Orf9b	QIQ–LAVTRME	18-27	QVQKPLSVTWMD	50
	SARS-CoV-2 RdRp/RNA complex	VQ–LSEISMD	168-176	VQKPLSVTWMD*	55
	SARS-CoV-2 RdRp/RNA complex	VIVNNLDKSA	494-503	VI-NNLTKSA*	80
**Measles**	Chain A, Spike glycoprotein, SARS-CoV-2	PSPGMPALLS	5-14	PSSTKPPALS*	50
	Chain A, Nucleoproprotein, SARS-CoV-2	SSSTKKSAAEAS	15-26	SSTTKSPASSAS	58
	Chain A, Nucleoproprotein, SARS-CoV-2	SNATKKSAAEAS	1-12	SSTTKSPASSAS*	50
	Chain A, Spike glycoprotein, SARS-CoV-2	TNSPRRAASVAS	678-689	TKSP—ASSAS	58
**Varicella zoster**	Chain B, Nucleoproprotein, SARS-CoV-2	TPGSSRGTSP	8-17	TPSEGRQPSP	50
	Chain A, Spike glycoprotein, SARS-CoV-2	PS-GRLVPRGSP	1210-1220	PSEGRQ-PSPSP*	58
	Chain A, Spike protein S2, SARS-CoV-2	RLQSLQTYVTQ	298-308	RLQDLSSCITQ*	55
	Chain N, Spike glycoprotein, SARS-CoV-2	GLPQGFSALE	215-224	GLPNFFRALE*	70
	Chain A, Spike glycoprotein, SARS-CoV-2	LRTRTQLPPA	18-27	LQT-TTLPPA*	70
	Chain A, Spike glycoprotein, SARS-CoV-2	QTQTNSPQQAQ	675-685	QTTTLPP–AQ	55
**Borrelia burgdor-feri**	Chain B, Spike protein S1, SARS-CoV-2	YNYLYRLFRLSNL	115-127	YNYL——SNL*	54
	Chain A, Spike glycoprotein, SARS-CoV-2	LS—TFLENLYFQGD-YK	1230-1244	LSSLTFL-NL–LGNPYK*	56
	Chain A, Spike glycoprotein, SARS-CoV-2	RKD-GEWVLLSTFLENL	1222-1237	RKDFAG—L-TFLEEL*	56
	Chain E, Spike protein S1, SARS-CoV-2	TGVLTESN—KKF	231-241	TG—ETNSLIKKF*	50
	Chain A, Spike glycoprotein, SARS-CoV-2	LS-ETKCTLKSFT	327-338	LSTGETNSLIKKFT*	50
	Chain A, Spike glycoprotein, SARS-CoV-2	ITNGSLEVLFQ	332-342	ITDES—LFQ	55
	Chain N, Spike glycoprotein, SARS-CoV-2	VFLV-LLPLVSSQ	3-14	VFLVPCLL—SQ*	57
	Chain A, Spike protein S2, SARS-CoV-2	SLQTYVTQQLI	301-311	SLQT—–LI*	55
	Chain A, Spike glycoprotein, SARS-CoV-2	LVDLPIGIN-ITRF	209-221	LV-LKISRNAITTF*	57
	Chain A, Spike glycoprotein, SARS-CoV-2	SFCTQLNRAL	777-786	SF-TQEQQAL*	60

* This subject sequence made a match with more than one section of the SARS-CoV-2 sequence. Only a selection of the overwhelming number of matches are shown in this table, with a cutoff of ID% of 50 and above.

**Table 2:** Potential cross-reactive epitopes between SARS-CoV-2 proteins and food antigens.


	SARS-CoV-2 antigen	SARS-CoV-2 sequence	Mapped start to end	Food sequence	ID (%)
**Peanut** **ARA H2 allergen**	The crystal structure of COVID-19 main protease in the apo state	TITVNVLAWLY–AA	197-209	TILV-ALA-LFLLAA*	53
	Chain A, Spike glycoprotein	LVSL-LSVLL	15-23	LVALALF-LL*	60
	Chain A, Spike glycoprotein	SQILPDPSKPS	777-787	SQ—DPYSPS*	55
	Chain A, Spike glycoprotein	SPS—–GAGSVASQ	699-709	SPSPYDRRGAGS–SQ*	56
	Replicase ORF1a polyprotein	LVA-EWFL-A	2323-2330	LVALALFLLA*	60
	Replicase ORF1a polyprotein	MPILTLTRAL	4634-4643	MAKLTILVAL	50
	Membrane glycoprotein	KLIFLWLLWPVTLACFVLAA	50-69	KLTIL—–VALALFLLAA	50
	Structure of Disulphide-stabilized SARS-CoV-2 Spike Protein Trimer (x2 disulphide-bond mutant, G413C, V987C, single Arg S1/S2 cleavage site)	CEAEVQI—–DRL	978-987	CEALQQIMENQSDRL	53
**α−gliadin**	Structure of SARS-CoV-2 3Q-2P full-length prefusion spike trimmer (C3 symmetry), SARS-CoV-2	PQ-QAQSVASQ	681-690	PQLQPQN-PSQ	55
	Chain A, Spike protein S2, SARS-CoV-2	QSLQTYVTQQ	300-309	QILQ—–QQ*	50
	Chain A, Spike protein S2, SARS-CoV-2	QQLIRAAEIRASANLA	308-323	QQLPQFEEIR—NLA*	56
	Chain A, Spike protein S1, SARS-CoV-2	SLV-SLLSVLL	14-23	SLVSSLVSMIL*	64
	Chain A, Spike protein S1, SARS-CoV-2	PALLSLV-SLLSVL	10-22	PAQLEVIRSL–VL*	50
**Glutenin**	Chain A, Spike glycoprotein, SARS-CoV-2	EVAKNLNESL	1185-1194	EVQANL–SL*	50
	Chain A, Spike protein S1, SARS-CoV-2	STNLVK–NKGSLE	215-226	STNLQKALSK-ALE	57
	Chain A, Nucleoprotein, SARS-CoV-2	DLKFPRG-QG	16-24	NLDFSKGHQG*	50
	Chain A, Spike glycoprotein, SARS-CoV-2	TQTNSPASVASQSI	676-689	TQTPTQAS-NSQFI	57
	Chain A, Spike glycoprotein, SARS-CoV-2	TRTQLPPAYTNS	20-31	TPTQ—A-SNS*	50
	Chain A, Spike glycoprotein, SARS-CoV-2	IQHSG–RPLESR	1246-1256	1HHPGAFPPLPSR*	54
**Rice endochiti-nase**	Chain A, Spike glycoprotein, SARS-CoV-2	RASANLAAIKIM	1019-1029	RALA-LAVVAM*	55
	Chain B Spike glycoprotein, SARS-CoV-2	RASANLAATKM	1111-1121	RALA-LAVVAM*	55
	Chain A, Spike glycoprotein, SARS-CoV-2	GGG—–SGGGSGGSS	1261-1272	GGGPTPPSSGGGSGVAS*	59
	Chain A, Spike glycoprotein, SARS-CoV-2	SGAGS-VASQSII	701-712	SGGGSGVA–SII*	69
	Chain R, Spike protein S1, SARS-CoV-2	CGGG——GSGSG	219-227	CGGGPTPPSSGGGSG	53
	Chain A, Spike glycoprotein, SARS-CoV-2	PSSA-SSVASQSII	681-693	PSSGGGSGVAS–II	60
	Chain A, Spike glycoprotein, SARS-CoV-2	SPGGSGSVASQSII	667-680	SSGGGSGVA–SII	57
	Chain A, Spike glycoprotein, SARS-CoV-2	GSGSGRVQPTESIV	1-14	GGGSG-V—ASII	50

**α-casein**	Chain A, Spike protein S2, SARS-CoV-2	LQSLQTYVTQQLIR	299-312	LQ-LQAAHAQEQIR*	50
**Dynorphin**	Chain A, Spike glycoprotein, SARS-CoV-2	TPSALGKLQD	915-924	TPSTLG-LND*	70
**Peanut agglutinin**	Chain A, Spike glycoprotein, SARS-CoV-2	TQCVNLTTRTQLPP	4-17	TQHPNVTT—LAP*	50
	Chain A, Spike glycoprotein, SARS-CoV-2	CSFGGVSVITP	564-574	CS—VSTATP*	55
	Chain A, Spike protein S2, SARS-CoV-2	FIAGLIAVLV	4-14	FIGG—IVLV	64
**Wheat germ agglutinin**	Chain B Spike glycoprotein, SARS-CoV-2	VLSHHFGKEL	72-81	VLSQKFEKEL	70
	Chain A, Spike glycoprotein, SARS-CoV-2	LVKQLSSNFG	53-62	LVIQLKESFG*	60
	Chain A, Spike glycoprotein, SARS-CoV-2	TGD—VNLTTRT	19-28	TGNIARVNLTTNT	62
	Chain A, Spike glycoprotein, SARS-CoV-2	PSAYTNSF-TRGV	25-36	PSA-SNAFMVCGV	54
**Lentil lectin**	Chain A, Spike protein S2, SARS-CoV-2	SLQTYVTQQLI	301-311	SLQT—QMI*	55
	Chain A, Spike glycoprotein, SARS-CoV-2	NGLTVLPPLLT	830-84	NVLTVT—LT*	55
**Pineapple bromelain**	Chain A, Spike glycoprotein, SARS-CoV-2	VASQSIIAYT	674-683	VA-Q—YT*	50
	Chain A, Spike protein S1, SARS-CoV-2	KRFDNPVLPFND	64-75	KR—EPVVSFDD*	50
	Chain A, Spike glycoprotein, SARS-CoV-2	KQGNFKNLSE	182-191	KRGNLVSLSE*	60
	Chain A, Spike protein S2, SARS-CoV-2	VTQQLIRAAEIRASAN	306-321	VSNQPI-AAALDASGN*	50
	Chain A, Spike protein S2, SARS-CoV-2	HFPREGVFVSN-GT	386-398	HYKR-GVFTGPCGT*	50
	Chain A, Nucleoprotein, SARS-CoV-2	QDPNSSSTKK	11-20	QD—SSGKK	60
**Almond allergen**	Chain E, Spike protein S1, SARS-CoV-2	FVF-LVLLPLV	2-11	FVFSLCLL-LV*	73
	Chain B, Spike glycoprotein, SARS-CoV-2	AFFFFLQLLGNVLV	57-70	AFVFSLCLL—LV	57
	Chain A, Spike protein S2, SARS-CoV-2	GINASVVNIQ	4-13	GVAASRITIQ*	50
	Chain A, Spike glycoprotein, SARS-CoV-2	QELGKYEQGSG	1188-1198	QE—QQGSG*	55
	Chain A, Spike protein S1, SARS-CoV-2	NENGTITDAVDCALD	268-282	NENG—DAI—LD*	53
	Chain A, Spike glycoprotein, SARS-CoV-2	YQTQTNSRRRAR	672-683	YQI—SREQAR*	50

* This subject sequence made a match with more than one section of the SARS-CoV-2 sequence. Only a selection of the overwhelming number of matches are shown in this table, with a cutoff of ID% of 50 and above.

## Discussion

In our earlier investigation, we applied anti-SARS-CoV-2 monoclonal antibody to 55 different tissue antigens and showed that these specific antibodies reacted with 28 autoantigens (20). We also sought selective peptide matches shared by spike protein and nucleoproteins with mitochondria M2, F-actin, and thyroid peroxidase, and found extensive cross-reactivity between them (20).

In another article in the same journal (15), Reche et al. explored potential cross-reactive immunity to SARS-CoV-2 from common human pathogens and vaccines. Among the tested 25 human pathogen and vaccine antigens, they found that viruses such as mumps, measles and rubella used in pediatric vaccinations did not contain SARS-CoV-2 cross-reactive epitopes, and concluded that immunity against these viruses may not provide any general protection against SARS-CoV-2. In comparison, the authors found combination vaccines against diphtheria, tetanus and pertussis (DTaP vaccine) to be significant sources of possible cross-reactive immunity to SARS-CoV-2 spike protein, which included numerous CD4, CD8, and B cell epitopes (15).

We were inspired by these findings, firstly, to investigate the reaction of SARS-CoV-2 monoclonal antibody with different pathogens and vaccines, particularly DTaP. Secondly, since in additional studies we showed evidence of immune reactivity by antibodies made against pathogens and human autoantigens towards different food antigens (22, 23, 26), we extended this current research to cross-reaction between SARS-CoV-2 proteins and foods that we consume on a daily basis. This is because it has been shown that the entry of undigested food antigens into the circulation results in the production of food-specific antibodies not just in individuals with disturbed microbiota and enhanced intestinal permeability but also in healthy subjects (23–33). The cross-reactive immunity elicited by food antigens and peptides in combination with bacterial, viral, and vaccine antigens is highly important since it may protect the general population against SARS-CoV-2 and other cross-reactive viruses (15, 27).

However, because epitope sharing between two proteins by itself is not necessarily an indication of immune cross-reactivity (34), based on our earlier experience (19, 20), we applied monoclonal antibodies made against SARS-CoV-2 spike protein and nucleoprotein to DTaP and BCG vaccine antigens, as well as to several common viruses and bacteria to which our immune system has most likely been exposed during our lifetime, to determine if in fact the shared epitopes and homology actually resulted in cross-reactions (16, 17, 35–37).

We chose to use human IgG_1_ monoclonal antibody made against SARS-CoV-2 spike protein S1 domain and human IgG_1_ monoclonal antibody made against SARS-CoV-2 nucleoprotein. We used rabbit polyclonal antibodies to react with different food antigens.

Why did we choose to focus mainly on monoclonal antibodies to study reactivity by SARS-CoV-2 with pathogens and vaccines? It is true that polyclonal antibodies have the ability to detect multiple epitopes on an antigen, giving them a higher overall affinity for their antigen and, therefore, greater detection efficiency. However, the heterogenous nature of polyclonal antibodies also makes them more prone to batch-to-batch variability and cross-reactivity with other molecules, resulting in a higher background. Monoclonal antibodies, on the other hand, only detect one epitope per antigen, thus reducing cross-reactivity with other molecules. This also reduces the possibility of false positives, which is precisely the reason why we chose to focus mainly on monoclonal antibodies (38, 39).

We chose this specific antibody from Novus because it binds specifically to amino acids 318-510 in the S (spike) domain of the SARS-CoV spike protein as well as the SARS-CoV-2 (COVID-19) spike protein, giving us a broad range with which to work. More importantly, this antibody binds to the receptor binding domain (RBD), which is the most important component of the viral spike glycoprotein that is found on SARS-CoV-2. The virus uses this spike protein to anchor itself to the ACE2 receptor on human cells before infecting them. The RBD ise used as an antigen to generate neutralizing antibodies that are used in monoclonal antibody therapy against the progression of COVID-19 (40, 41).

Likewise, we used human SARS-Cov-2 nucleoprotein monoclonal antibody because it recognizes and binds the non-linear/conformational epitope of the N protein of SARS-CoV and SARS-CoV-2. This protein, as opposed to other viral components (S1, S2), can induce innate memory in human primary monocytes. This innate memory from viral nucleoproteins may contribute to the overall response to viral or bacterial infections or the response to vaccination (42).

In this present study, we found that SARS-CoV-2 spike protein specific antibody reacted most significantly with DTaP vaccine, to a somewhat lesser degree with *E. faecalis* bacteria which commonly resides in the human gut, to even lesser degrees with EBV-EAD, EBNA, HSV 1 + 2 CMV, and *B. burgdorferi*, but not significantly at all with BCG, measles, *H. influenza*, EBV-VCA, HHV-6, VZV, *E. coli* CdT and LPS (**Figure 1**). While the reaction of SARS-CoV-2 nucleoprotein monoclonal antibody with DTaP vaccine was the strongest, the overall reaction of anti-nucleoprotein antibody with the vaccines, viral and bacterial antigens was less strong (**Figure 2**). These results further confirm the findings of Reche (15), that the combination of DTaP vaccines are significant sources of T and B cell cross-reactivity to SARS-CoV-2, and cross-reactive immunity to DTaP vaccines can be protective against SARS-CoV-2.

BCG is a live attenuated strain of *M. bovis* used against tuberculosis. It has been shown that BCG can elicit protective heterologous immunity to SARS-CoV-2 (43–47). This protection of BCG and the induction of heterologous protective immunity against SARS-CoV-2 and other viruses is explained by the induction of trained immunity and functional reprogramming of innate immunity (15, 48–50). This conclusion was based on the observation that countries that implement BCG vaccination have fewer COVID-19 cases (43–46). Thus, we were consequently surprised when our purchased monoclonal antibodies made against both SARS-CoV-2 spike protein and nucleoprotein did not react with BCG vaccine antigens. In fact, the OD obtained from these reactions was comparable to the ELISA background, or less than 0.3. The lack of immune reactivity by SARS-CoV-2 spike protein and nucleoprotein antibodies with BCG may show that the presence of cross-reactive epitopes between two proteins does not necessarily result in cross-reactive immunity (34). However, our own results may be due to our use of human SARS-CoV-2 spike protein monoclonal antibody that binds specifically to amino acids 318-510 in the S domain of the SARS-CoV and SARS-CoV-2 spike proteins, and to our use of human SARS-Cov-2 nucleoprotein monoclonal antibody which recognizes and binds the non-linear/conformational epitope of the N protein of SARS-CoV and SARS-CoV-2. We admit that it is all too possible that BCG may be reacting with one or more of the many other SARS-CoV-2 epitopes different from the ones we used, such as the non-structural proteins also shown in our tables. Interestingly, while studying potential cross-reactivity between SARS-CoV-2 and BCG, Reche found 120, out of which 41 were for B-cell epitopes, 21 were for CD8 epitopes, and 11 were for CD4 epitopes (15).

Regarding common bacteria and viruses, including herpesviruses, we found significant reactivity by monoclonal antibody to spike protein with *E. faecalis* and moderate reactions with some of the herpes family of viruses (**Figure 1**). These reactions with this enterobacter, EBV and HSV 1 + 2 may be significant, because IgG antibody against these pathogens is found in various degrees in the blood of the general population (51). More research is needed on whether or not these common bacteria and viruses can be protective against SARS-CoV-2.

Because in our earlier studies we had shown that antibodies specific to both SARS-CoV-2 and food reacted with a variety of human tissue antigens (19–28, 30–32), in this study we hypothesized that many food proteins and peptides may share homology with SARS-CoV-2 proteins, and thus immune reaction against food proteins may be protective against SARS-CoV-2 infection. To test this hypothesis, we applied monoclonal antibody against SARS-CoV-2 spike protein to 180 different food proteins or peptides. This resulted in a significant reaction with 28 foods and weaker reactions with 2 foods, while immune reactivity with the other 150 food resulted in ODs of less than 0.56 or the mean ± 3SD of all the non-reactive foods, the ODs of which were very close to the background. Milk, α+β casein, gliadin toxic peptide, soy, pea protein, roasted almond, lentil lectin and other commonly consumed foods were among those that reacted with SARS-CoV-2 spike protein antibody (**Figure 3**). With SARS-CoV-2 nucleoprotein antibody the reaction was strongest with broccoli, roasted almond, cashew, soy bean, rice endochitinase, pork, pineapple bromelain, and gliadin toxic peptide. The reaction between SARS-CoV-2 nucleoprotein antibody and 20 other foods, although significant, was not as strong (**Figure 4**). Additional experiments performed in this research supports the hypothesis that this anti-SARS-CoV-2-specific antibody reaction with common vaccine antigens (DTaP), bacteria (*E. faecalis*), common viruses (EBV, HSV 1 + 2), and food proteins such as α+β casein, gliadin peptide, pea protein and others is specific:

1. Reaction of anti-SARS-CoV-2 protein antibodies resulted in a significant decline in antibody reaction with vaccine, viral and food antigens in proportion to their dilutions (**Figures 5**, **6**)
2. Reaction of affinity-purified polyclonal antibodies made against different foods with SARS-CoV-2 spike protein and nucleoprotein resulted in a significant reaction only if the anti-food antibodies were made against the food items with which SARS-CoV-2 antibody had reacted in the earlier experiment (**Figures 5**–**7**). Rabbit polyclonal antibodies were used because monoclonal antibodies for these foods are not available.
3. Only human sera that had high levels of IgG antibodies to herpesviruses and food antigens such as gliadin, milk, α+β casein and pineapple bromelain, reacted significantly with SARS-CoV-2 spike protein (**Figure 8**)
4. We found significant homology between SARS-CoV-2 proteins and vaccine antigens as well as common viruses shown previously (15) and in this study (**Tables 1**, **2**), and between SARS-CoV-2 proteins and different food items shown for the first time in this manuscript

Finally, we would like to admit that at this level we don’t know if these cross-reactive antibodies produced against a virus like EBV or foods like gliadin or α+β casein are protective or not against SARS-CoV-2. Considering the phenomenon that has been referred to as the “original antigenic sin” (17, 52), “whereby a history of a response to cross-reactive antigens can bias the response towards those antigens and inhibit the response to a new infection or vaccine” (17, 52), we should interpret our results with caution. Especially, since, very recently in different articles, it was shown that not only is EBV DNA increased in COVID-19 patients, but EBV reactivation and lytic replication induces ACE2 expression and enhances SARS-CoV-2 entry into the epithelial cell (53–55). Additionally, antibody cross-reactivity between casein and myelin associated glycoprotein (MAG) was shown to result in central nervous system demyelination (56). Thus, we do not definitively know if cross-reactive antibodies produced against viral and food epitopes that share similarity with SARS-CoV-2 proteins are helpful in controlling SARS-CoV-2 infections.

## Conclusion

The findings presented in this manuscript indicate that cross-reactivity elicited by DTaP vaccines in combination with common herpesviruses to which we are exposed at an early age, bacteria that are part of our normal flora (*E. faecalis, E. coli*), and food that we consume on a daily basis may be keeping some individuals safe from COVID-19 in different parts of the world. This cross-reactivity between different pathogens and food antigens may explain why a significant percentage of the population who were repeatedly exposed to different variants of SARS-CoV-2 never became seriously ill. Additional *in vivo* and *in vitro* experiments are needed to clarify whether or not this cross-protection was due to the presence of cross-reactive antibodies or long-term memory T and B cells in the blood. Although cross-reactivity is mainly viewed as negative, this cross-reaction involving vaccine antigens, common viruses and food antigens may be protective.

## Limitations of the study

- We admit that there are several limitations to our study.
- We applied human monoclonal antibodies made against SARS-CoV-2 spike protein and nucleoprotein in some experiments but used purified rabbit polyclonal antibodies in others due to lack of availability. Upon their availability, experiments should be performed comparing different clones of monoclonal antibodies and different preparations of polyclonal antibodies, testing their reactivity with different vaccines, pathogens and food proteins.
- We studied only a limited number of vaccines, bacteria and viruses in comparison to 180 different food antigens for the presence of cross-reactive immunity.
- Due to the high costs of SARS-CoV-2 recombinant antigens, for determining the specificity of antibody-antigen reaction we performed only serial dilutions and did not perform inhibition studies, which require high concentrations of antigens
- We used the BLAST sequence matching program to study the degrees of possible amino acid sequence homology shared by SARS-CoV-2 proteins with different viruses and food antigens, but not with T- and B-cell cross-reactive epitopes, as was done by Reche (15).

## Data availability statement

The original contributions presented in the study are included in the article/**Supplementary Material**. Further inquiries can be directed to the corresponding author.

## Ethics statement

We purchased human monoclonal and rabbit polyclonal antibodies from certified, regulated commercial sources who use immunization protocols for the animals that conform to The Guide for the Care and Use of Laboratory Animals published by the National Institutes of Health, publication no 85-23, 1985. Human sera from healthy donors were obtained from commercial sources and were screened for presence or absence of antibodies against different viral and food antigens.

## Authors contributions

AV, EV, and JR designed the study. AV, EV, and AM performed the ELISA assays. AM and JR did the BLAST search. AV and EV wrote the manuscript, and the other authors read and approved it. All authors contributed to the article and approved the submitted version.

## Funding

All funding was provided by Immunosciences Lab., Inc., of which the corresponding author, Aristo Vojdani, is co-owner, CEO, and technical director.

## Acknowledgments

The authors wish to thank Joel Bautista for making the graphs, editing the manuscript for English corrections, and typing the manuscript for submission.

## Conflict of interest

Corresponding author Aristo Vojdani is the co-owner, CEO and Technical Director of Immunosciences Lab., Inc. He is also the Chief Scientific Advisor of Cyrex Labs, LLC on a consultancy basis.

The remaining authors declare that the research was conducted in the absence of any commercial or financial relationships that could be constructed as a potential conflict of interest.

## Publisher’s note

All claims expressed in this article are solely those of the authors and do not necessarily represent those of their affiliated organizations, or those of the publisher, the editors and the reviewers. Any product that may be evaluated in this article, or claim that may be made by its manufacturer, is not guaranteed or endorsed by the publisher.

## References

[1] Huang C Wang Y Li X Ren L Zhao J Hu Y. Clinical features of patients infected with 2019 novel coronavirus in wuhan, China. Lancet (2020) 395:497–506. doi: 10.1016/S0140-6736(20)30183-5 31986264 PMC7159299

[2] Le Bert N Tan AT Kunasegaran K Tham CYL Hafezi M Chia A. SARS-CoV-2-specific T cell immunity in cases of COVID-19 and SARS and uninfected controls. Nature (2020) 584(7821):457–62. doi: 10.1038/s41586-020-2550-z 32668444

[3] Grifoni A Weiskopf D Ramirez SI Mateus J Dan JM Moderbacher CR. Targets of T cell responses to SARS-CoV-2 coronavirus in humans with COVID-19 disease and unexposed individuals. Cell (2020) 181(7):1489–501. doi: 10.1016/j.cell.2020.05.015 PMC7237901 32473127

[4] Huibin LV Wu NC Tsang OTY Yuan M Perera RAPM Leung WS. Cross-reactive antibody response between SARS-CoV-2 and SARS-CoV infections. Cell Rep (2020) 31(9):107725. doi: 10.1016/j.celrep.2020.107725 PMC7231734 32426212

[5] Sette A Crotty S. Pre-existing immunity to SARS-CoV-2: the knowns and unknowns. Nat Rev Immunol (2020) 20:457–8. doi: 10.1038/s41577-020-0389-z PMC7339790 32636479

[6] Mateus J Grifoni A Tarke A Sidney J Ramirez SI Dan JM. Selective and cross-reactive SARS-CoV-2 T cell epitopes in unexposed humans. Science (2020) 370(6512):89–94. doi: 10.1126/science.abd3871 32753554 PMC7574914

[7] Quiros-Fernandez I Poorebrahim M Fakhr E Cid-Arregui A. Immunogenic T cell epitopes of SARS-CoV-2 are recognized by circulating memory and naive CD8 T cells of unexposed individuals. EBioMedicine (2021) 72:103610. doi: 10.1016/j.ebiom.2021.103610 34627082 PMC8493415

[8] Meckiff BJ Ramirez-Suástegui C Fajardo V Chee SJ Kusnadi A Simon H. Imbalance of regulatory and cytotoxic SARS-CoV-2-reactive CD4^+^ T cells in COVID-19. Cell (2020) 183(5):1340–53. doi: 10.1016/j.cell.2020.10.001 PMC7534589 33096020

[9] Weiskopf D Schmitz KS Raadsen MP Grifoni A Okba NMA Endeman H. Phenotype and kinetics of SARS-CoV-2-specific T cells in COVID-19 patients with acute respiratory distress syndrome. Sci Immunol (2020) 5(48):eabd2071. doi: 10.1126/sciimmunol.abd2071 32591408 PMC7319493

[10] Braun J Loyal L Frentsch M Wendisch D Georg P Kurth F. SARS-CoV-2-reactive T cells in healthy donors and patients with COVID-19. Nature (2020) 587:270–4. doi: 10.1038/s41586-020-2598-9 32726801

[11] Nickbakhsh S Ho A Marques DFP McMenamin J Gunson RN Murcia PR. Epidemiology of seasonal coronaviruses: Establishing the context for the emergence of coronavirus disease 2019. J Infect Dis (2020) 222:17–25. doi: 10.1093/infdis/jiaa185 32296837 PMC7184404

[12] Kissler SM Tedijanto C Goldstein E Grad YH Lipsitch M. Protecting the transmission dynamics of SARS-CoV-2 through the postpandemic period. Science (2020) 368:860–8. doi: 10.1126/science.abb5793 PMC7164482 32291278

[13] Lipsitch M Grad YH Sette A Crotty S. Cross-reactive memory T cells and herd immunity to SARS-CoV-2. Nat Rev Immunol (2020) 20(11):709–13. doi: 10.1038/s41577-020-00460-4 PMC7537578 33024281

[14] Grifoni A Sidney J Vita R Peters B Crotty S Weiskopf D. SARS-CoV-2 human T cell epitopes: Adaptive immune response against COVID-19. Cell Host Microbe (2021) 29(7):1076–92. doi: 10.1016/j.chom.2021.05.010 PMC8139264 34237248

[15] Reche PA. Potential cross-reactive immunity to SARS-CoV-2 from common human pathogens and vaccine. Front Immunol (2020) 11:586984. doi: 10.3389/fimmu.2020.586984 33178220 PMC7596387

[16] Agrawal B. Heterologous immunity: Role in natural and vaccine-induced resistance to infections. Front Immunol (2019) 10:2631. doi: 10.3389/fimmu.2019.02631 31781118 PMC6856678

[17] Welsh RM Fujinami RS. Pathogenic epitopes, heterologous immunity and vaccine design. Nat Rev Microbiol (2007) 5(7):555–63. doi: 10.1038/nrmicro1709 PMC7097378 17558423

[18] Gu XX Plotkin SA Edwards KM Sette A Mills KHG Levy O. Waning immunity and microbial vaccines – workshop of the national institute of allergy and infectious diseases. Clin Vaccine Immunol (2017) 24(7):e00034–17. doi: 10.1128/CVI.00034-17 PMC5498725 28490424

[19] Vojdani A Kharrazian D. Potential antigenic cross-reactivity between SARS-CoV-2 and human tissue with a possible link to an increase in autoimmune diseases. Clin Immunol (2020) 217:108480. doi: 10.1016/j.clim.2020.108480 32461193 PMC7246018

[20] Vojdani A Vojdani E Kharrazian D. Reaction of human monoclonal antibodies to SARS-CoV-2 proteins with tissue antigens: Implications for autoimmune diseases. Front Immunol (2021) 11:61708919. doi: 10.3389/fimmu.2020.617089 PMC7873987 33584709

[21] Vojdani A Tarash I. Cross-reaction between gliadin and different food and tissue antigens. Food Nutr Sci (2013) 44:20–32. doi: 10.4236/fns.2013.41005

[22] Vojdani A Vojdani E. Immunoreactivity of anti-AβP-42 specific antibody with toxic chemicals and food antigens. J Alzheimers Dis Parkinsonism (2018) 8(3):441. doi: 10.4172/2161-0460.1000441

[23] Vojdani A Turnpaugh CC. Antibodies against group a streptococcus, dopamine receptors, and ganglioside GM1 cross-react with a variety of food antigens, potentially interfering with biomarkers for PANS and PANDAS. Biomarkers Neuropsychiatry (2020) 3:100023. doi: 10.1016/j.bionps.2020.100023

[24] Vojdani A Gushgari LR Vojdani E. Interaction between food antigens and the immune system: Association with autoimmune disorders. Autoimmun Rev (2020) 19(3):102459. doi: 10.1016/j.autrev.2020.102459 31917265

[25] Vojdani A. Reaction of food-specific antibodies with different tissue antigens. Int J Food Sci Tech (2020) 55(4):1800–15. doi: 10.1111/ijfs.14467

[26] Vojdani A Lerner A Vojdani E. Cross-reactivity and sequence homology between alpha-synuclein and food products: A step further for parkinson's disease synucleinopathy. Cells (2021) 10(5):1111. doi: 10.3390/cells10051111 34063062 PMC8147930

[27] Vojdani A Monro J Lanzisera F Sadeghi H. Serological cross-reactivity between viruses and their contribution to autoimmunity. Autoimmun Rev (2021) 20:102840. doi: 10.1016/j.autrev.2021.102840 33971342

[28] Kharrazian D Herbert M Vojdani A. Detection of islet cell immune reactivity with low glycemic index foods – is this a concern for type 1 diabetes? J Diabetes Res (2017) 2017:4124967. doi: 10.1155/2017/4124967 28819632 PMC5551512

[29] Riccio P Rossano R. Undigested food and gut microbiota may cooperate in the pathogenesis of neuroinflammatory diseases: A matter of barriers and a proposal on the origin of organ specificity. Nutrients (2019) 11:2714. doi: 10.3390/nu11112714 PMC6893834 31717475

[30] Hvatum M Kanerud L Hallgren R Brandtzaeg P. The gut-joint axis: Cross-reactive food antibodies in rheumatoid arthritis. Gut (2006) 55:1240–7. doi: 10.1136/gut.2005.076901 PMC1860040 16484508

[31] Vojdani A Mukherjee PS Berookhim J Kharrazian D. Detection of antibodies against human and plant aquaporins in patients with multiple sclerosis. Autoimmun Dis (2015) 2015:905208. doi: 10.1155/2015/905208 PMC4529886 26290755

[32] Vojdani A. Molecular mimicry as a mechanism for food immune reactivities and autoimmunity. ATHM (2015) 21:34–45. 25599184

[33] Vojdani A Vojdani E Kharrazian D. Fluctuation of zonulin levels in blood versus stability of antibodies. World J Gastroenterol (2017) 23:5669–79. doi: 10.3748/wjg.v23.i31.5669 PMC5569281 28883692

[34] Lee CH Salio M Napolitani G Ogg G Simmons A Koohy H. Predicting cross-reactivity and antigen-specificity of T cell receptors. Front Immunol (2020) 11:565096. doi: 10.3389/fimmu.2020.565096 33193332 PMC7642207

[35] Mosca A LeClerc M Hugot JP. Gut microbiota diversity and human diseases: Should we reintroduce key predators in our ecosystem? Front Microbiol (2016) 7:455. doi: 10.3389/fmicb.2016.00455 27065999 PMC4815357

[36] Carrasco Pro S Lindestam Arlehamn CS Dhanda SK Carpenter C Lindvall M Faruqi AA. Microbiota epitope similarity either dampens or enhances the immunogenicity of disease-associated antigenic epitopes. PLoSOne (2018) 13:e0196551. doi: 10.1371/journal.pone.0196551 PMC5937769 29734356

[37] Belkaid Y Hand TW. Role of the microbiota in immunity and inflammation. Cell (2014) 157:121–41. doi: 10.1016/j.cell.2014.03.011 PMC4056765 24679531

[38] Lipman NS Jackson LR Trudel LJ Weis-Garcia F. Monoclonal versus polyclonal antibodies: distinguishing characteristics, applications, and information resources. ILAR J (2005) 46:258–68. doi: 10.1093/ilar.46.3.258 15953833

[39] Busby M Xue C Li C Farjoun Y Gienger E Yofe I. Systemic comparison of monoclonal versus polyclonal antibodies for mapping histone modifications by ChIP-seq. Epigenet Chromatin (2016) 9:49. doi: 10.1186/s13072-016-0100-6 PMC5097419 27826357

[40] Makdasi E Levy Y Alcalay R Noy-Porat T Zahavy E Mechaly A. Neutralizing monoclonal anti-SARS-CoV-2 antibodies isolated from immunized rabbits define novel vulnerable spike-protein epitope. Viruses (2021) 13:566. doi: 10.3390/v13040566 33810465 PMC8065470

[41] Min L Sun Q. Antibodies and vaccines target RBD of SARS-CoV-2. Front Mol Biosci (2021) 8:671633. doi: 10.3389/fmolb.2021.671633 33968996 PMC8100443

[42] Urban P Italiani P Boraschi D Gloria S. The SARS-CoC-2 nucleoprotein induces innate memory in human monocytes. Front Immunol (2022) 13:963627. doi: 10.3389/fimmu.2022.963627 35928816 PMC9343583

[43] Miller A Reandelar MJ Fasciglione K Roumanova V Li Y Otazu GH. Correlation between universal BCG vaccination policy and reduced morbidity and mortality for COVID-19: an epidemiological study. medRxiv (2020). doi: 10.1101/2020.03.24.20042937

[44] Curtis N Sparrow A Ghebreyesus TA Netea MG. Considering BCG vaccination to reduce the impact of COVID-19. Lancet (2020) 396:1546–6. doi: 10.1016/S0140-6736(20)31025-4 PMC7252177 32359402

[45] O’Neill LAJ Netea MG. BCG-Induced trained immunity: can it offer protection against COVID-19? Nat Rev Immunol (2020) 20:335–7. doi: 10.1038/s41577-020-0337-y PMC7212510 32393823

[46] Wickramasinghe D Wickramasinghe N Kamburugamuwa SA Arambepola C Samarasekera N. Correlation between immunity from BCG and the morbidity and mortality of COVID-19. Trop Diseases Travel Med Vaccines (2020) 6:17. doi: 10.1186/s40794-020-00117-z 32868985 PMC7453689

[47] Mangtani P Abubakar I Ariti C Beynon R Pimpin L Fine PE. Protection by BCG vaccine against tuberculosis: a systematic review of randomized controlled trials. Clin Infect Dis (2014) 58:470–80. doi: 10.1093/cid/cit790 24336911

[48] Arts RJW Carvalho A La Rocca C Palma C Rodrigues F Silvestre R. Immunometabolic pathways in BCG-induced trained immunity. Cell Rep (2016) 17:2562–71. doi: 10.1016/j.celrep.2016.11.011 PMC5177620 27926861

[49] Moorlag S Arts RJW van Crevel R Netea MG. Non-specific effects of BCG vaccine on viral infections. Clin Microbiol Infect (2019) 25:1473–8. doi: 10.1016/j.cmi.2019.04.020 31055165

[50] Sanchez-Ramon S Conejero L Netea MG Sancho D Palomares I Subiza JL. Trained immunity-based vaccines: A new paradigm for the development of broad-spectrum anti-infectious formulations. Front Immunol (2018) 9:2936. doi: 10.3389/fimmu.2018.02936 PMC6304371 30619296

[51] Smatti MK Yassine HM AbuOdeh R Almarawani A Taleb SA Althani AA. Prevalence and molecular profiling of Epstein Barr virus (EBV) among healthy blood donors from different nationalities in Qatar. PloS One (2017) 12(12):e0189033. doi: 10.1371/journal.pone.0189033 29228016 PMC5724864

[52] Zhang A Stacey HD Mullarkey CE Miller MS. Original antigenic sin: How first exposure shapes lifelong anti-influenza virus immune responses. J Immunol (2019) 202(2):335–40. doi: 10.4049/jimmunol.1801149 30617114

[53] Paoilucci S Cassaniti I Novazzi F Fiorina L Pirallas A Comolli G. San Matteo pavia COVID-19 task force. EBV DNA increase in COVID-19 patients with impaired lymphocyte subpopulation count. Int J Infect Dis (2021) 104:315–9. doi: 10.1016/j.ijid.2020.12.051 PMC7833117 33359064

[54] Gold JE Okyay RA Licht WE Hurley DJ. Investigation of long COVID prevalence and its relationship to Epstein-Barr virus reactivation. Pathogens (2021) 10(6):763. doi: 10.3390/pathogens10060763 34204243 PMC8233978

[55] Verma D Church TM Swaminathan S. Epstein-Barr Virus lytic replication induces ACE2 expression and enhances SARS-CoV-2 pseudotyped virus entry in epithelial cells. J Virol (2021) 95(13):e0019221. doi: 10.1128/JVI.00192-21 33853968 PMC8316011

[56] Chunder R Weier A Maurer H Kuerten S. Antibody cross-reactivity between casein and myelin associated glycoprotein results in central nervous system demyelination. PNAS (2022) 119(10):e2117034119. doi: 10.1073/pnas.2117034119 35235454 PMC8916005

