# Supplementary material for: Reaction of SARS-CoV-2 antibodies with other pathogens, vaccines, and food antigens
Source: Front Immunol. 2022 Sep 23;13:1003094. doi: 10.3389/fimmu.2022.1003094 (PMC9537454; doi:10.3389/fimmu.2022.1003094)
Supplement: Supplementary file 1 [file DataSheet_1.docx]

The 180 Foods

1. Egg White cooked
2. Egg Yolk cooked
3. Goat`s Milk
4. Soft Cheese + Hard Cheese
5. Yogurt
6. Rice white + brown cooked
7. Rice Cake
8. Rice Protein
9. Rice Endochitinase
10. Wild Rice cooked
11. Wheat + Alpha-Gliadins
12. Black Bean cooked
13. Bean Agglutinins
14. Dark Chocolate + Cocoa
15. Fava Bean cooked
16. Garbanzo Bean cooked
17. Kidney Bean cooked
18. Lentil cooked
19. Lentil Lectin
20. Lima Bean cooked
21. Pinto Beans cooked
22. Soy Sauce gluten-free
23. Soybean Agglutinin
24. Soybean Oleosin + Aquaporin
25. Tofu
26. Almond
27. Almond roasted
28. Brazil Nut raw + roasted
29. Cashew
30. Cashew roasted
31. Cashew Vicilin
32. Chia Seed
33. Flax Seed
34. Hazelnut raw + roasted
35. Macadamia Nut raw + roasted
36. Mustard Seed
37. Peanut roasted
38. Pecan raw + roasted
39. Peanut Butter
40. Peanut Agglutinin
41. Peanut Oleosin
42. Pistachio raw + roasted
43. Pumpkin Seeds roasted
44. Sesame Oleosin
45. Sunflower Seeds roasted
46. Sesame Albumin
47. Walnut
48. Artichoke cooked
49. Asparagus cooked
50. Asparagus
51. Beet cooked
52. Bell Pepper
53. Broccoli
54. Broccoli cooked
55. Brussels Sprouts cooked
56. Cabbage red + green
57. Canola Oleosin
58. Carrot
59. Cauliflower cooked
60. Celery
61. Chili Pepper
62. Cabbage red + green cooked
63. Carrot cooked
64. Corn + Aquaporin cooked
65. Corn Oleosin
66. Cucumber pickled
67. Eggplant cooked
68. Garlic
69. Garlic cooked
70. Green Bean cooked
71. Lettuce
72. Mushroom raw+ cooked
73. Onion+ Scallion
74. Okra cooked
75. Olive green + black pickled
76. Onion + Scallion cooked
77. Pea cooked
78. Pea Protein
79. Pea Lectin
80. Potato white cooked (fried)
81. Potato white cooked (cooked)
82. Popped Corn
83. Pumpkin+ Squash cooked
84. Radish
85. Spinach+ Aquaporin
86. Safflower + Sunflower Oleosin
87. Seaweed
88. Tomato + Aquaporin
89. Tomato Paste
90. Yam+ Sweet Potato cooked
91. Zucchini cooked
92. Apple
93. Apple Cider
94. Apricot
95. Avocado
96. Banana
97. Banana cooked
98. Blueberry
99. Cantaloupe + Honeydew Melon
100. Cherry
101. Cranberry
102. Coconut meat + water
103. Date
104. Fig
105. Grape red + green
106. Grapefruit
107. Kiwi
108. Lemon + Lime
109. Latex Hevein
110. Mango
111. Orange Juice
112. Orange
113. Peach + Nectarine
114. Pear
115. Pineapple
116. Papaya
117. Pineapple Bromelain
118. Plum
119. Pomegranate
120. Red Wine
121. Strawberry
122. Watermelon
123. White Wine
124. Cod cooked
125. Crab + Lobster cooked
126. Clam cooked
127. Halibut cooked
128. Imitation Crab cooked
129. Mackerel cooked
130. Oyster cooked
131. Parvalbumin
132. Red Snapper cooked
133. Salmon
134. Salmon cooked
135. Sardine + Anchovy cooked
136. Sea Bass cooked
137. Shrimp cooked
138. Shrimp Tropomyosin
139. Scallops cooked
140. Squid (Calamari) cooked
141. Tuna cooked
142. Tilapia cooked
143. Trout cooked
144. Tuna raw
145. Whitefish cooked
146. Beef cooked medium
147. Chicken cooked
148. Gelatin
149. Lamb cooked
150. Meat Glue
151. Turkey cooked
152. Pork cooked
153. Basil
154. Cilantro
155. Cumin
156. Dill
157. Mint
158. Oregano
159. Parsley
160. Rosemary
161. Thyme
162. Cinnamon
163. Ginger
164. Clove
165. Nutmeg
166. Paprika
167. Turmeric (Curcumin)
168. Vanilla
169. Carrageenan
170. Beta-Glucan
171. Gum Guar
172. Gum Tragacanth
173. Mastic Gum + Gum Arabic
174. Locust Bean Gum
175. Xanthan Gum
176. Black Tea brewed
177. Coffee Bean Protein brewed
178. Green Tea brewed
179. Food Coloring
180. Honey raw + processed
